# Metabolomics reveals differences of metal toxicity in cultures of *Pseudomonas pseudoalcaligenes* KF707 grown on different carbon sources

**DOI:** 10.3389/fmicb.2015.00827

**Published:** 2015-08-17

**Authors:** Sean C. Booth, Aalim M. Weljie, Raymond J. Turner

**Affiliations:** ^1^Department of Biological Sciences, University of Calgary, CalgaryAB, Canada; ^2^Department of Systems Pharmacology and Translational Therapeutics, Smilow Centre for Translational Research, Perelman School of Medicine, University of Pennsylvania, PhiladelphiaPA, USA; ^3^Biofilm Research Group, University of Calgary, CalgaryAB, Canada

**Keywords:** bacteria, metal toxicity, *Pseudomonas*, GC-MS metabolomics, bioremediation, aluminum, copper, biphenyl

## Abstract

Co-contamination of metals and organic pollutants is a global problem as metals interfere with the metabolism of complex organics by bacteria. Based on a prior observation that metal tolerance was altered by the sole carbon source being used for growth, we sought to understand how metal toxicity specifically affects bacteria using an organic pollutant as their sole carbon source. To this end metabolomics was used to compare cultures of *Pseudomonas pseudoalcaligenes* KF707 grown on either biphenyl (Bp) or succinate (Sc) as the sole carbon source in the presence of either aluminum (Al) or copper (Cu). Using multivariate statistical analysis it was found that the metals caused perturbations to more cellular processes in the cultures grown on Bp than those grown on Sc. Al induced many changes that were indicative of increased oxidative stress as metabolites involved in DNA damage and protection, the Krebs cycle and anti-oxidant production were altered. Cu also caused metabolic changes that were indicative of similar stress, as well as appearing to disrupt other key enzymes such as fumarase. Additionally, both metals caused the accumulation of Bp degradation intermediates indicating that they interfered with Bp metabolism. Together these results provide a basic understanding of how metal toxicity specifically affects bacteria at a biochemical level during the degradation of an organic pollutant and implicate the catabolism of this carbon source as a major factor that exacerbates metal toxicity.

## Introduction

Anthropogenic pollution in the form of organic compounds and metal elements is widespread around the globe (Darnault et al., 2005; Zalasiewicz et al., 2011). Bioremediation is the process of using living organisms to either degrade organic pollutants into innocuous end-products or immobilize metals and is an excellent method for cleaning up pollution and preventing ecological damage (Shukla et al., 2010). While many sites contaminated with just organic pollutants have been successfully remediated, co-contamination of metals has been found to interfere with bacterial degradation of organic pollutants (Sandrin and Hoffman, 2007). This is especially problematic as a large proportion of sites (e.g., 40% of U.S. E.P.A. superfund sites) are contaminated with both types of pollutants (Sandrin et al., 2000). Despite this issue, little of the research on understanding mechanisms of metal toxicity in bacteria has focused on this problem (Lemire et al., 2013). Additionally, applied research has emphasized only the characterization of situations where various metals inhibited the degradation of different pollutants (Olaniran et al., 2013) while the underlying physiological effects of these metals on bacteria have yet to be investigated thoroughly.

Past work on metal toxicity in bacteria was mainly focused on determining the concentration of metals that inhibited growth, but these concentrations were found to vary widely depending on the growth medium as this determines the speciation and therefore bioavailability of metals (Sandrin et al., 2000). While investigating this issue of how media composition affects metal toxicity, we observed that tolerance to metals also differed depending on the sole carbon source provided for growth, all other media components being equivalent (Booth et al., 2013a). As cells growing on different carbon sources make use of different metabolic pathways, it was postulated that the cellular targets of metal toxicity could differ based on the carbon source being used. Previously we used metabolomics to uncover differences between how surface attached biofilms of *Pseudomonas fluorescens* and free-swimming planktonic cultures were affected by copper (Cu) exposure (Booth et al., 2011b). This systems biology technique enables the identification and quantification of the low-molecular weight compounds within a sample, thereby providing a metabolic profile. As metabolomics has been successfully applied to understanding metal toxicity in a variety of prokaryotic and eukaryotic systems (Booth et al., 2011a), here we sought to use metabolomics to characterize the differential effects of metal toxicity in bacterial cultures grown on a either simple carbon source or a model pollutant.

*Pseudomonas pseudoalcaligenes* KF707 is a bacterium that was first studied due to its ability to degrade biphenyl (Bp) and polychlorinated biphenyls (Furukawa and Miyazaki, 1986; Taira et al., 1992) and has since been studied with regards to its metal-resistance capabilities (Tremaroli et al., 2008, 2009), chemotaxis toward Bp (Tremaroli et al., 2010) and recently had its genome sequenced (Triscari-Barberi et al., 2012). Compared to other metals and bacteria, *P. pseudoalcaligenes* KF707 was found to be more sensitive to Cu and aluminum (Al; Tremaroli et al., 2010) but was able to tolerate higher concentrations of these metals when grown on succinate (Sc) compared to Bp (Booth et al., 2013a). Both of these metals have been found in co-contamination with polycyclic aromatic hydrocarbons and polychlorinated biphenyls (Allen, 2008; Burgess et al., 2009; Renzi et al., 2009; Jartun and Pettersen, 2010; Annicchiarico et al., 2011; Hassanvand et al., 2015) especially in electronic waste (Robinson, 2009; Fornalczyk et al., 2013; Liu et al., 2013; Itai et al., 2014; Pradhan and Kumar, 2014) their mechanisms of toxicity have been characterized in other systems (Macomber et al., 2007; Macomber and Imlay, 2009; Lemire et al., 2010; Mailloux et al., 2011) and are physicochemically very distinct from one-another (Lemire et al., 2013). As such, these metals were selected to determine if the physiological effects of metal toxicity were the same in cultures grown on different carbon sources. To this end we used gas-chromatography mass-spectrometry (GC-MS) metabolomics to characterize cultures and spent media of *P. pseudoalcaligenes* KF707 grown on either Sc or Bp, in the presence of the same, sub-inhibitory concentrations of either Al or Cu. By comparing metabolic profiles using multi-variate statistical techniques, differences were discovered in how the carbon source being used for growth influenced the effects of Al and Cu. To our knowledge this provides the first systems-wide characterization of the combined effects of metal toxicity and growth on an aromatic carbon source. Our results indicate that Bp catabolism is both affected by and exacerbates metal toxicity as multiple metabolic pathways were altered in response to this combined stress. These insights into the physiological effects of metal toxicity in an environmentally isolated bacterium should provide a basis for further investigations into the biochemical mechanisms of how metal toxicity disrupts the metabolism of complex aromatic substrates.

## Experimental Procedures

### Culture Growth

*Pseudomonas pseudoalcaligenes* KF707 was routinely cultured in minimal salts medium (MSM) consisting of (in g/L) K_2_HPO_4_, 4.4; KH_2_PO_4_, 1.7; (NH_4_)_2_SO_4_, 2.6; MgSO_4_⋅7H_2_O, 0.4; CaSO_4_⋅2H_2_O, 0.0031; MnSO_4_⋅H_2_O, 0.05; FeSO_4_⋅7H_2_O, 0.1 (Tremaroli et al., 2010). Trace metals were filter sterilized using a 0.2 μm filter and added as a 20X stock directly to each culture flask. Eight percent dimethyl sulfoxide (DMSO) frozen stocks were used to inoculate 5 mL subcultures which were grown overnight. Fifty micro liter (Sc) or 1 mL (Bp) was then used to inoculate 250 mL flasks containing 50 mL of MSM with either 5 mM Sc or 0.39 g of sterile Bp as this compound is insoluble in water. At the same time as inoculation Al was added in the form of Al_2_(SO_4_)_3_ or Cu as CuSO_4_ to a final concentration of 3 mM or 60 μM (respectively). One of each culture type was grown simultaneously five separate times for five replicates of each condition. For growth curves, every 8 h two aliquots of 50 μL were removed and separately serially diluted 1/10 down to 10^-7^. Twenty micro liter spots were plated on LB agar and counted after 24 h of incubation at 30°C. For pH determination, separate cultures were grown and every 8 h 1 mL of culture was removed, centrifuged for 5 min at 10,000 RPM and the pH determined using a Beckman 720 pH meter and probe (Beckman, Pasadena, CA, USA).

### Collection of Samples and Extraction of Metabolites

All cultures were treated identically for sample collection and metabolite extraction, except that Bp-grown cultures were poured through a coarse filter to strain out residual Bp particles. After 24 h of growth cultures were harvested by rapid centrifugation at 4°C for 5 min at 5,000 RPM in a Sorvall RC5B-plus using the SLA-1500 rotor (Thermo Scientific, Waltham, MA, USA). Supernatant was collected as ‘Spent Media’ and all samples were immediately frozen in liquid nitrogen for storage at -80°C. After all samples were collected, metabolites were extracted. The extraction solution (900 μL 2:1 methanol:chloroform) was added directly to frozen cell pellets. After homogenization by pipetting the sample was transferred to a 2 mL MpBio FastDNA Spin kit vial. Cells were lysed via bead-beating according to the manufacturer’s instructions (40 s at 6.0 power level; MP Biomedicals, Santa Ana, CA, USA). Samples were returned to ice immediately and processed as previously (Booth et al., 2011b). Briefly, 300 μL water and chloroform were added to all samples. After hand-mixing, samples were centrifuged for 7 min at 14,000 RPM and the aqueous phase was transferred to a fresh tube. These steps were repeated twice to obtain pure aqueous samples which were dried down in a vacuum concentrator at room temperature and stored until analysis at -80°C. For spent media samples, 1.5 mL thawed sample was dried down at room temperature in a vacuum concentrator. After resuspension in 50 μL ddH_2_O, 900 μL 2:1 methanol:chloroform was added and samples were treated identically to cellular samples post bead beating. For extraction/derivitization controls, clean, empty vials were treated identically to true samples.

### Derivitization and Analysis by GC-MS

Sample derivitization and GC-MS analysis was performed identically to our previous work (Booth et al., 2011b; Bhat et al., 2015) according to (Gullberg et al., 2004). Fifty micro liter of 20 mg/mL methoxylamine in pyridine was added to samples which were mixed and incubated for 2 h at 37°C, 200 RPM. Next, 50 μL of *N*-methyl-*N*-(trimethylsilyl)trifluoroacetamide (MSTFA, Sigma Aldrich, St. Louis, MO, USA) was added to all samples which were incubated identically for 45 min. Samples were then diluted with hexane and centrifuged for 7 min at 14,000 RPM to remove particulates. 150 μL was transferred to gastight vials for analysis on a Waters GCT premier mass spectrometer. Helium was used as the carrier gas at a constant flow of 1.2 ml min^-1^. One micro liter derivatized sample was injected into a DB5-MS column (splitless, 30m × 0.25 mm ID × 0.25 μm) at an injector temperature of 275°C. Initial column temperature was 80°C which was held for 1 min and then ramped at 12°C min^-1^ to 320°C and held for 8 min. The MS was operated in a range of 50–800 m/z.

### Identification of Metabolites

Mass spectral deconvolution, calibration, identification and analysis were performed using Automated Mass Spectral Deconvolution and Identification Software (AMDIS; Stein, 1999). Data were first calibrated for retention time shifts using a set of alkane standards (C10–C30). The GOLM Metabolome Database (GMD; Hummel et al., 2007) VAR5 library was imported into the NIST MS Search program (Stein, 2011) and components were identified and confirmed manually using both the GMD library and the NIST11 Mass Spectral library. A custom library of reproducible but unidentifiable analytes was also generated from components extracted from representative samples using AMDIS. For details see supplementary material.

### Removal of Derivatization Artifacts

Any analytes found in the extraction/derivatization controls that contained no sample were considered artifactual and were excluded from quantification. These compounds originated either from derivatization reactions occurring between the plastics of the sample vessel, solvents, or from the GC column. The most biologically relevant compounds that were removed from quantification were uracil, decanoic, dodecanoic, hexadecanoic, heptadecanoic, and octadecanoic acid. For a detailed list of compounds see the supplementary material.

### Quantification of Metabolites

Concurrent to the generation of a library of mass spectra for the identification of components, ions were selected from each compound as representatives for quantification. These ions were manually selected to ensure that they were unique to the retention index window of the analyte and of high intensity relative to all other fragmentation ions. The common trimethyl-silyl (TMS) ions 73, and 147 as well as ions with high background such as 121, 266, 285, and 299 were excluded from selection to ensure that the ions being quantified were truly representative of the analyte in question. Peak quantification was performed using this ion retention time list using MET-IDEA (Broeckling et al., 2006). Peak selection parameters were manually tuned (for specifics, see supplementary material) to ensure that quantification was representative of individual analytes. After quantification the validity of each analyte’s ions as representative of that analyte was determined. Using a custom R script (R-Project for Statistical Computing, CRAN.R-project.org) the correlation across all samples between all pairs of ions as well as the sum of all quantified ions was determined for each analyte, for details see supplementary material. Any ion with a correlation to the sum of all ions from that analyte below 0.8 was removed from subsequent analysis. This process excluded some analytes from further analysis as they had no ions that passed this threshold. Most notable was cysteine 3TMS. The remaining ions were summed for each analyte. Any analytes that represented the same metabolite (i.e., aspartate 2TMS and aspartate 3TMS) were summed. This gave 269 metabolites quantified, of which 89 were identified and used for subsequent analysis.

### Statistical Analysis: Pre-Processing

Data were first processed to enable their proper downstream analysis. A noise threshold was determined by calculating the mean intensity of analytes in the extraction control samples. Any value below this mean was interpreted as noise and was thus set to zero. Data were then normalized by probabilistic quotient normalization (PQN; Dieterle et al., 2006) in order to account for any variation in cellular material collected, despite cell densities being highly similar between control and treated samples. This kind of normalization was preferred as past experiences attempting to normalize to sample wet-weight or total integral normalization were inferior (Booth et al., 2011b). For details on the normalization procedure, see the supplementary material. After normalization data were log transformed and each analyte was mean-centered and scaled to unit-variance. These transformations allow variables with disparate dynamic ranges and means to be compared on the same scale. Comparison of sample clustering between raw, log transformed data (Supplementary Figure S1) and normalized data (Supplementary Figure S2) indicated that the normalization did not unduly skew the dataset as clustering remained similar.

### Statistical Analysis

Statistical analyses were performed using SIMCA P+ v13.0 (UMETRICS) and R (R-Project for Statistical Computing, CRAN.R-project.org). PCA was performed on all samples together as well as individual analyses for each class. This separate analysis was used to remove any outlying samples that were not highly similar to the remaining members of that class. Only a single sample was removed by this process, for details see the supplementary material. After this trimming process PCA and OPLS-DA was performed on the entire dataset. OPLS-DA models were then generated for each combination of control and treated samples (e.g., Bp control and Bp Al exposed, Sc control spent media and Sc Cu exposed spent media etc.) in order to minimize the amount of variation being examined in any one model, thereby maximizing the interpretability of each model. For each of these pairwise OPLS-DA models the R_2_Y, Q_2_ and CV-ANOVA p values were used to assess model quality; only models with CV-ANOVA p values below 0.05 were accepted as statistically significant. From each significant model the VIP and *p*(corr) values were exported for further interpretation. Shared and unique structures plots were used as this type of plot simplifies analysis of metabolomics data while maintaining the depth of complexity within the dataset (Wiklund et al., 2008).

### Identification of Unknown Analytes

Biological interpretation of the data implied the possibility of several metabolites that had not been identified, and could not be identified due to their lack of standards available in either Golm or NIST libraries. Using manual fragmentation to predict the mass spectra, all the intermediates of the two possible catechol-degrading pathways (starting either with catechol 1,2-dioxygenase or catechol 2,3-dioxygenase) were searched for, as well as several other metabolites that were predicted to be present. Only two compounds with unknown peaks that matched to a high enough thresholds were found. From these predicted analytes their functional groups were used to predict the Kováts’ retention index (Stein et al., 2007). This approach confidently identified two unknown analytes: 2-hydroxymuconic semialdehyde and 2-phosphoglycolic acid. For details of the identification procedure see the supplementary material.

### Pathway Enrichment Analysis

mBROLE was used to determine which metabolic pathways were being affected by metal toxicity in cultures grown on the two carbon sources. By calculating the number of metabolites affected that occur in a particular pathway mBROLE can determine which metabolic pathways were most affected under a condition (Chagoyen and Pazos, 2011). From the OPLS-DA models comparing control and metal exposed cells for each carbon source and metal, metabolites with a VIP > 0.8 were submitted using their KEGG IDs (Ogata et al., 1999). Pathways that were enriched with a false discovery rate adjusted *p*-value <0.05 were accepted as affected by metal exposure. Metabolic pathways that are commonly found by this type of analysis due to overbroad interpretation of KEGG pathways were removed. For details see supplementary material.

## Results and Discussion

### Growth of *P. pseudoalcaligenes* KF707 in the Presence of Metals on Succinate and Biphenyl

**FIGURE 1:**
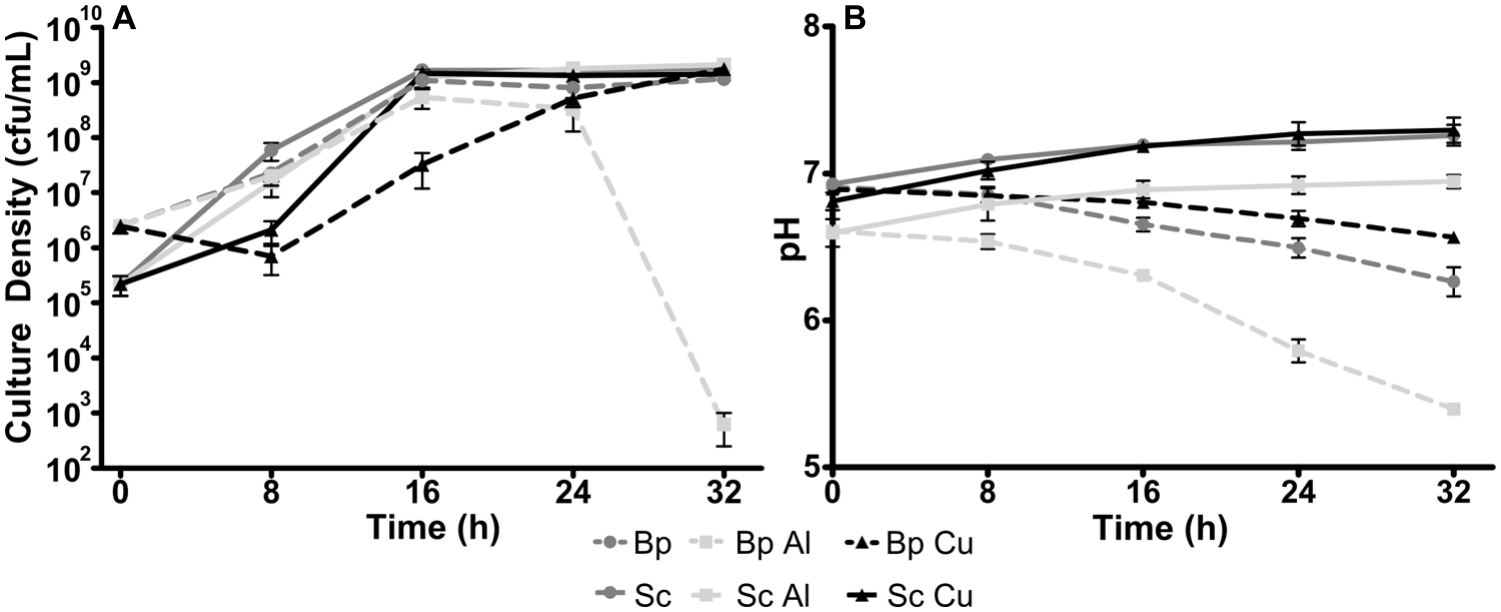
**Growth **(A)** and culture pH **(B)** of *Pseudomonas pseudoalcaligenes* KF707 grown over 32 h in minimal salts medium (MSM) with either succinate (Sc) or biphenyl (Bp) as the sole carbon source, with either nothing, 3 mM Al_2_(SO_4_)_3_ (Al) or 60 μM CuSO_4_ (Cu).** Points denote the mean of 3 biological replicates, error bars indicate SEM.

The minimum concentrations of Al and Cu that inhibited the growth of *P. pseudoalcaligenes* KF707 using Sc or Bp as the sole carbon source were previously determined using high-throughput microtitre plate assays (Booth et al., 2013a). To confirm that these concentrations were relevant in the larger cultures needed for metabolomics, culture growth in the presence of these metals was quantified over time. Based on our prior work (Booth et al., 2013a), 3 mM Al and 60 μM Cu was selected as metal concentrations that would elicit a phenotype but not inhibit growth. The higher concentration of Al was used to overcome the lack of bioavailability of Al caused by the phosphate in the medium. Phosphate was used as a buffer despite its well-characterized property of chelating Al (Berthon, 2002). However, we could not use organic buffering agents such as MOPS as this would have negated the single carbon source nature of the study. To confirm that these concentrations did not inhibit growth in 250 mL flasks, cultures were grown for 32 h in the presence of either 3 mM Al or 60 μM Cu. After 24 h of growth, the numbers of viable cells from metal-exposed cultures were found to be similar to their control counterparts for both carbon sources (**Figure 1A**). As comparable cell densities and growth period were desirable to make comparisons between control and metal exposed cultures as similar as possible, 24 h was thus selected as the time point for metabolomics harvest.

Culture pH at each time point was also determined. Over 32 h, the pH of the culture medium from Bp-grown cells decreased about 1 unit for Al exposed cultures and 0.5 units for control and Cu exposed (**Figure 1B**). The pH of Sc-grown cultures did not change in such a manner. During GC-MS metabolomic characterization, large quantities of benzoic acid were found in the cells and spent media of all samples grown on Bp. The amounts were so great that it could not be quantified comparably to other metabolites that were detected as the GC-MS detector was saturated. The only other metabolite that was saturated was phosphate, the buffering agent from the medium. Benzoic acid is produced during catabolism of Bp (Furukawa and Fujihara, 2008), making it an unavoidable byproduct. The decrease in pH observed in Bp grown cultures (**Figure 1B**) was likely due to this acid being produced. As pH decreases from neutral, Al is known to increase in solubility, which is considered one of the main problems of acid rain (Macdonald and Bruce Martin, 1988). The Bp-grown Al exposed cultures’ viability decreased sharply from 24 to 32 h as the pH dropped from about 6 to 5.5. This low of a pH would have increased the bioavailablity of Al, evidently to the point of lethal toxicity. As Al is prevalent throughout the Earth’s crust (Exley, 2009), the degradation of organic compounds in sites with low pH would be expected to be more difficult as increased bioavailability of Al would result in greater stress to bacteria.

### Metabolomic Characterization of Cultures

**FIGURE 2:**
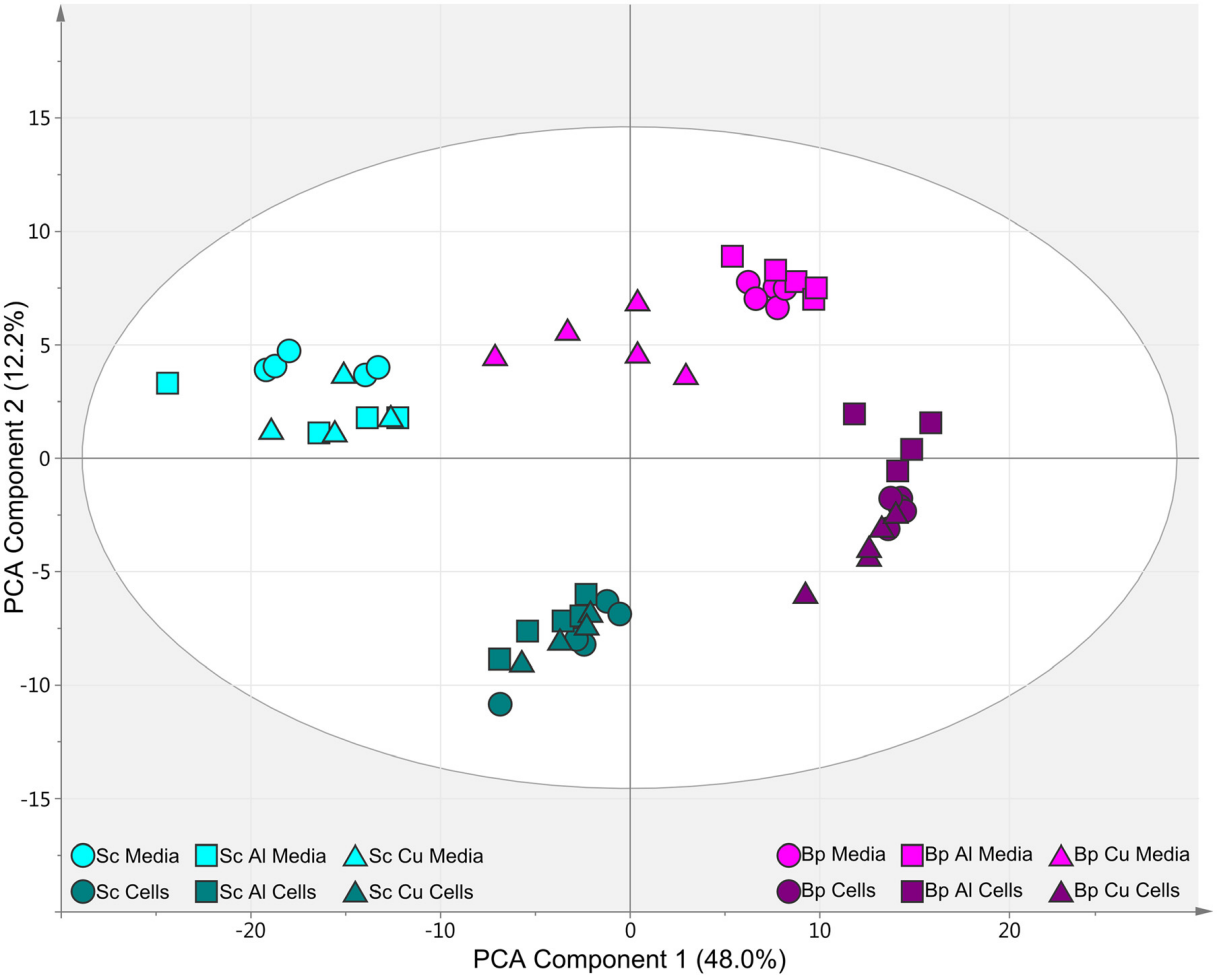
**Principal component analysis scores plot of GC-MS metabolite profiles of cells and spent media from cultures of *P. pseudoalcaligenes* KF707 grown on either biphenyl (Bp, purple) or succinate (Sc, teal) as the sole carbon source and exposed to either control (circles), 3 mM Al (squares) or 60 μM Cu (triangles).** Results were normalized, scaled and centered before analysis.

To understand how exposure to Al or Cu affected bacterial cultures, untreated samples were compared to those grown in the presence of each metal. GC-MS metabolic profiles were obtained from cells and spent media from cultures grown either on Sc or Bp as the sole carbon source and exposed separately to each metal. After exclusion of low quality analytes and artifactual compounds derived from reactions between the derivatization agents and plastics of the sample vessels, 269 metabolites were quantified, of which 89 were identified. These data were analyzed by the unsupervised statistical techniques hierarchical clustering analysis (HCL) and PCA. Inspection of the PCA scores plot (**Figure 2**) revealed that samples separated first by carbon source and next by sample type (cells or spent media). This model had a good *R*^2^ (0.741, variance explained) and *Q*^2^ (0.643, goodness of fit; **Table 1**), indicating that close to 75% of the variation in the dataset could be explained by carbon source and sample type. These overall trends were confirmed by HCL of the raw, log-transformed data (Supplementary Figure S1) and normalized, scaled data (Supplementary Figure S2). Both techniques showed that after being separated into groups of the same sample type and carbon source, there were still differences between control and metal exposed samples. While these analyses demonstrated that the metabolic profiles of samples varied based on the treatments applied, discerning specific changes to metabolites based on these treatments was non-trivial. In order to better understand the relation of specific metabolites to the altered conditions, we further extended the multivariate analysis using supervised techniques.

**Table 1:** Model statistics from PCA and pairwise OPLS-DA models comparing normalized, centered and scaled metabolite abundances in cells and spent media from *Pseudomonas pseudoalcaligenes* KF707 grown on either succinate (Sc) or biphenyl (Bp) with or without aluminum (Al) or copper (Cu).

Model	Type	Components	*R*^2^	*Q*^2^	CV-ANOVA *p*-value
All	PCA	5	0.741	0.643	NA
All	OPLS-DA	7 + 0	0.577	0.404	<0.001
Biphenyl Al	OPLS-DA	1 + 1	0.993	0.931	0.014
Biphenyl Cu	OPLS-DA	1 + 1	0.999	0.945	0.002
Biphenyl Al Media	OPLS-DA	1 + 0	0.821	0.647	0.026
Biphenyl Cu Media	OPLS-DA	1 + 1	0.989	0.952	0.002
Succinate Al	OPLS-DA	1 + 1	0.996	0.895	0.012
Succinate Cu	OPLS-DA	1 + 0	0.944	0.667	0.037
Succinate Al Media	OPLS-DA	1 + 1	0.989	0.897	0.029
Succinate Cu Media	OPLS-DA	1 + 1	0.996	0.916	0.020

*Components indicates number of predictive and orthogonal components present in the model, *R^2^* indicates cumulative variance accounted for by the model, *Q*^2^ cumulative variance predicted by the model and CV-ANOVA *p*-value was obtained from sevenfold cross-validation analysis of variance. Models with a *p*-value <0.05 were considered significant.*

### Supervised Statistical Analysis

**FIGURE 3:**
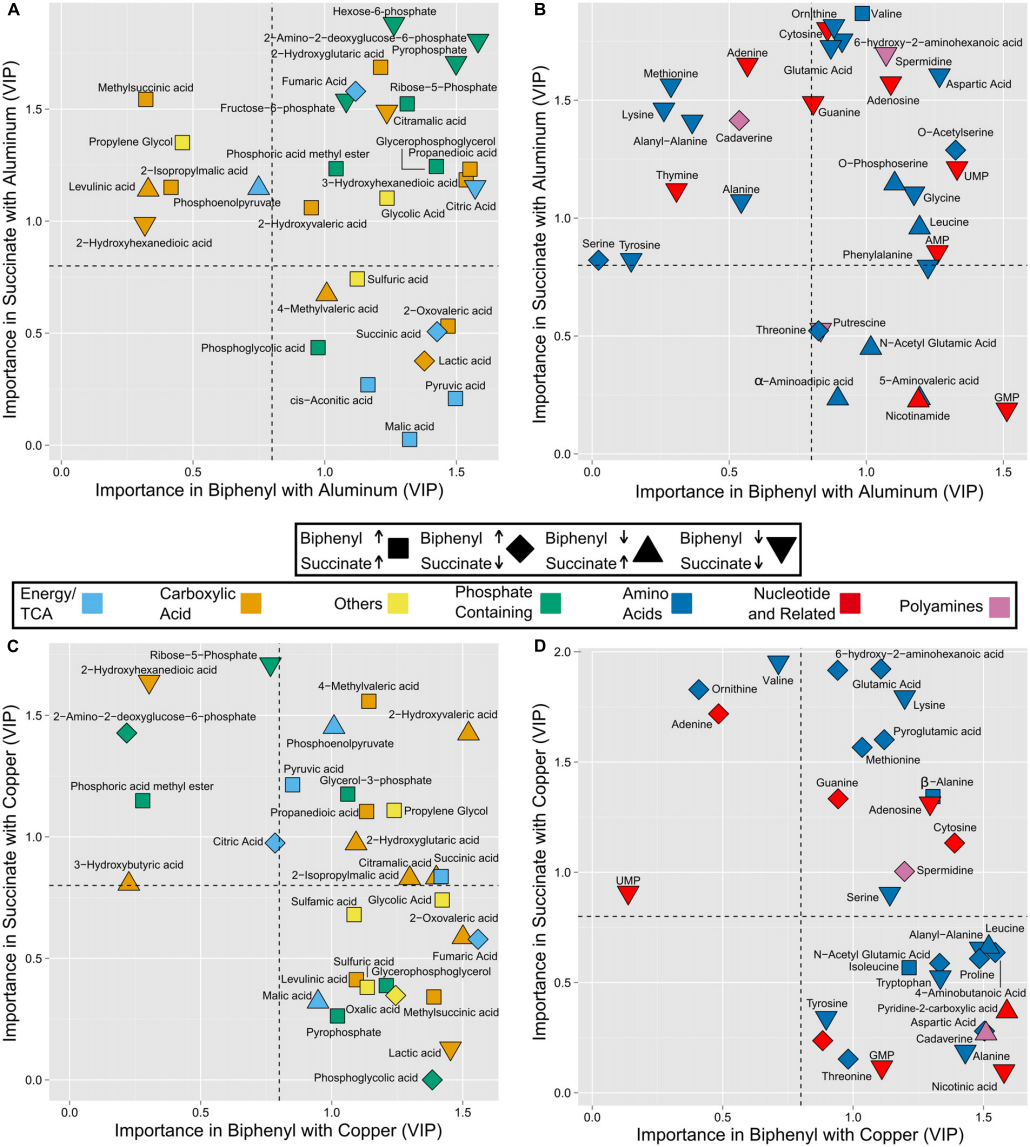
**Shared and unique structures plots showing comparison of changes to intracellular metabolites caused by Al **(A,B)** and Cu **(C,D)** in cultures of *P. pseudoalcaligenes* KF707 grown on either Sc or Bp as the sole carbon source.** Coordinates were determined by the VIP of each metabolite, as obtained from OPLS-DA models comparing control and metal exposed samples for either Sc (*y*-axes) or Bp (*x*-axes). Metabolites with a VIP ≥ 0.8 (dashed lines) indicate a significant change occurred in the metal exposed samples, those that were below in both cases were omitted. The association of each metabolite with control or metal exposed samples was determined using *p*(corr), which indicates the degree of correlation of the metabolite with a sample type. Shapes were assigned that indicate how the metabolite was altered by metal exposure: increased in both Bp and Sc (squares), increased in Bp but decreased in Sc (diamonds), increased in Sc but decreased in Bp (triangle up) and decreased in both (triangle down).

Pairwise OPLS-DA models were used to identify exactly which metabolites were being altered by metal exposure and how. These models compared just the control and one type of metal exposed sample in each carbon source for each sample type, allowing for the relative concentrations of metabolites to be compared between the control and treated samples. This eased interpretability and avoided confounding influences caused by carbon source and sample type. Examination of the statistics for each of these models revealed that they all accounted for most of the variation between samples (*R*^2^ ≥ 82%) as well as predicted the vast majority of variation (all but two models *Q*^2^ ≥ 89%) (**Table 1**; Supplementary Figures S4 and S5). Additionally the sevenfold cross-validation analysis of variance (CV-ANOVA) *p*-value, which essentially indicates the probability that such a model would be generated by chance, was <0.05 for all models. As in past studies (Booth et al., 2011b; Bhat et al., 2015) the pairwise OPLS-DA models showed significant differences between control and treated samples, so from these models the VIP and *p*(corr) were extracted. These values respectively indicate the importance of a metabolite in distinguishing the sample classes (i.e., control from metal exposed) and whether it is correlated with the control or metal exposed samples [for exact details on *p*(corr), see the supplementary material]. These data were subsequently used to produce shared and unique structures plots (Wiklund et al., 2008) for intracellular metabolites (**Figure 3**) as well as those that were found within the spent medium (Supplementary Figure S7) to determine similarities and differences between how metabolites were altered in each carbon source.

### Pathway Enrichment Analysis

Pathway enrichment analysis was used to identify metabolic pathways that were affected under each condition. Lists of metabolites that were identified as changing significantly between control and metal exposed samples (based on their VIP) from each model were separately submitted to mBROLE (Chagoyen and Pazos, 2011). This tool uses the annotations from the KEGG (Ogata et al., 1999) to determine which metabolic pathways a metabolite is involved in. Pathways are assigned *p*-values based on the probability of enough closely connected metabolites from the same pathway being altered only by random chance. After exclusion of spurious pathways (Booth et al., 2013b), many pathways were found to be affected by Al and Cu exposure in cultures of *P. pseudoalcaligenes* KF707 grown on Bp (**Table 2**). Fewer pathways were affected in the cultures grown on Sc. The most pathways were affected in Bp-grown cultures exposed to Cu, whereas Sc-grown cultures had the least number of pathways affected. This confirmed our expectation that metal toxicity would affect cultures differently depending on the carbon source being used. The pathways identified by mBROLE were subsequently used to contextualize the meaning of changes to individual metabolites and interpret why these metabolites were altered under each condition.

**Table 2:** Metabolic pathways affected by metal toxicity in cultures of *Pseudomonas pseudoalcaligenes* KF707 grown on either Bp or Sc as the sole carbon source in the presence of Al or Cu as determined by mBROLE.

Pathway	Bp Al	Sc Al	Bp Cu	Sc Cu
Benzoate degradation (via hydroxylation)	<0.01	NA	<0.01	NA
C5-Branched dibasic acid metabolism	0.02	NA	0.06	0.05
(Glycolysis/)Gluconeogenesis	NA	NA	0.06	0.05
Pentose phosphate pathway	0.09	NA	NA	0.05
Citrate cycle (Krebs cycle)	<0.01	0.01	<0.01	<0.01
Pyruvate metabolism	NA	0.02	<0.01	<0.01
Glyoxylate/dicarboxylate metabolism	<0.01	NA	<0.01	0.02
Pantothenate/CoA-biosynthesis	NA	NA	0.06	0.05
Purine metabolism	<0.01	<0.01	0.03	0.02
Nicotinate/nicotinamide metabolism	0.03	NA	0.02	NA
beta-Alanine metabolism	0.09	0.08	<0.01	0.01
Alanine, aspartate, and glutamate metabolism	<0.01	0.06	<0.01	<0.01
Arginine/proline metabolism	<0.01	NA	0.00	NA
Glycine, serine, and threonine metabolism	0.01	0.03	0.03	0.09
Valine, leucine, and isoleucine biosynthesis	0.01	0.07	<0.01	0.05
Cysteine/methionine metabolism	0.01	0.04	0.04	0.11
Sulfur metabolism	0.04	0.04	0.03	NA

*Presented here are modified *p*-values (i.e., multiple-testing corrected) indicating the probability that a pathway was affected under a particular condition. All reported pathways had an unmodified *p*-value <0.05 in at least one sample type. Metabolites were selected based on their VIP > 0.8 as determined from OPLS-DA models comparing control to metal exposed cultures and were submitted to mBROLE. Only metabolites with a known KEGG ID were used. *p*-values are colored according to siginificance: unmodified > 0.05, not siginificant (black), unmodified < 0.05 but modified > 0.05 (gray), unmodified and modified < 0.05 (white). Pathways are grouped into carbon metabolism (white), purine/pyrimidine metabolism (light gray), amino acid metabolism (gray) and sulfur containing metabolism (dark gray).*

### Toxicity Effects of Aluminum

Aluminum exposure caused similar alterations to many phosphate containing metabolites in cells grown on both carbon sources (**Figure 3A**, green symbols). Phosphate is a strong chelator of Al (Berthon, 2002). In *Rhizobium* species, increased production of extracellular polymeric substances was correlated with increased tolerance to Al (Ferreira et al., 2012). The decrease of phosphorylated sugars observed here could indicate their use in generating EPS with functional groups for binding Al, similarly to our past observation that metabolites involved in EPS production were increased in biofilm cultures of *P. fluorescens* exposed to Cu (Booth et al., 2011b). Alternatively the sugar-phosphates could be being used in lipopolysaccharide (LPS) synthesis as these outer membrane molecules could prevent Al entry into the cell by chelation (Silipo and Molinaro, 2010). Our results here indicate that phosphate containing EPS or LPS mediated protection of cells from metal stress may be used both by planktonic and biofilm cultures of *Pseudomonas.*

Ribose-5-phosphate (R5P) was increased in both carbon sources under Al stress. When exposed to Al, *P. fluorescens* was previously observed to increase NADPH production, partially via overexpression of glucose-6-phosphate (G6P) dehydrogenase (Singh et al., 2005). This enzyme catalyzes the first step in the PPP of which the oxidative portion uses ATP to generate NADPH and ends with R5P (Wood, 1986). The PPP was identified by mBROLE, but was not considered significant (**Table 2**). Still, the observed accumulation of R5P, and depletion of fructose-6-phosphate and hexose-6-phosphate (representative of G6P, see supplementary material) thus suggests that the oxidative portion of the PPP was being used to generate NADPH in response to oxidative stress being caused by Al. Conversely to R5P, malic acid and pyruvic acid were only accumulated in Bp-grown cultures (**Figure 3A**, light blue symbols). These two metabolites were previously observed to be increased when *P. fluorescens* was subject to oxidative stress from menadione as part of a metabolic network aimed at converting NADH to NADPH (Singh et al., 2008). In this network, pyruvate was increased to generate oxaloacetic acid, which was converted to malic acid in order to oxidize NADH to NAD and the malic acid was cleaved to produce pyruvic acid and reduce NADP to NADPH. The metabolomic results found here indicate that a similar metabolic network was thus likely active in *P. pseudoalcaligenes* KF707 cultures grown on Bp and exposed to Al, but not those grown on Sc. Additionally, pyruvate accumulation could be due to the ability of pyruvate to react and detoxify hydrogen peroxide (Giandomenico et al., 1997), making its accumulation a potentially useful anti-oxidant strategy. Pyruvate and malic acid were not affected by Al stress when cultures were grown on Sc, implying that the PPP sufficient for anti-oxidant production under these conditions.

Glycolic acid was increased in both carbon sources (**Figure 3A**, yellow square). This was an unexpected metabolite as it is produced by very few metabolic reactions (Ogata et al., 1999). One reaction is the dephosphorylation of phosphoglycolic acid. As this metabolite was not present in the libraries used for identification, it was manually identified from the unknown metabolites (see Supplementary Material for details). Thus it was observed that 2-phosphoglycolic acid was increased only in Bp-grown cultures (**Figure 3A**, green square), which was surprising as this metabolite also has few precursors and is associated generally with carbon fixation (Shively et al., 1998). More pertinently, when hydroxyl radicals react with the 4′ carbon of a ribose moiety of DNA, repair of this oxidative damage results in the production of 2-phosphoglycolic acid (Kuznetsova et al., 2009). Subsequent cleavage by phosphoglycolate phosphatase allows the salvage of the phosphate and two-carbon glycolic acid (Pellicer et al., 2003). Thus the accumulation of these two unexpected metabolites implies that Al is exerting toxicity by oxidatively damaging DNA, especially under Bp degrading conditions. Al has been well characterized as a pro-oxidant (Exley, 2004) while normal aerobic metabolism produces the ROS superoxide (O_2_^∙^), peroxide (H_2_O_2_) and hydroxyl radicals (OH^∙^) by incidental reactions between molecular oxygen (O_2_) and electron transport chain components (Imlay, 2003). It has been hypothesized that hydrated Al complexes stabilize superoxide radicals (which has received recent support (Mujika et al., 2014)), and this complex can then reduce Fe(III) to Fe(II), regenerating the active Al(III)-superoxide complex. Fe(II) undergoes the Fenton reaction with H_2_O_2_ generated from aerobic metabolism to produce 2 OH^∙^ radicals (Stohs and Bagchi, 1995). These radicals could then go on to react with DNA, causing the aforementioned accumulation of metabolites. These mechanisms were likely active and responsible for DNA damage which was repaired to produce 2-phosphoglycolic acid. *P. pseudoalcaligenes* KF707 possess a phosphoglycolic acid phosphatase, however, based on a BLAST search it surprisingly does not have any of the genes encoding for any subunits of glycolate oxidase (Triscari-Barberi et al., 2012). This explains the accumulation of glycolic acid as it was generated from oxidative DNA damage but cannot be re-assimilated into central carbon metabolism. Further indicating that oxidative stress caused DNA damage, nucleobases and nucleotides were decreased in response to Al, the specifics depending on carbon source (**Figure 3B**, red symbols) and purine metabolism was implicated by mBROLE (**Table 2**). In addition to oxidative stress that can damage the ribose moiety, the bases of DNA can be affected by ROS (Alberts et al., 2002). Repairing this damage requires all four nucleotides, though only AMP and GMP were detected. The individual bases were also detected and decreased, implying that they were being used up generating nucleotides for use in repairing DNA.

Further similarities were observed between *P. pseudoalcaligenes* KF707 and *P. fluorescens* exposed to Al. Under Al stress *P. fluorescens* also modifies its Krebs cycle to produce less NADH and more NADPH by using the glyoxylate cycle to shunt carbon from isocitrate to succinyl-CoA (Singh et al., 2009). This metabolic pathway produces both oxalate and glyoxylate, of which only the former was detected in this experiment. As in *P. fluorescens*, oxalate may have been secreted to chelate Al, but such an increase in secretion was only detected in Sc grown cultures (Supplementary Figure S6A). Alternatively, this pathway may not have been a viable option in *P. pseudoalcaligenes* growing on Bp as the accumulation of *cis*-aconitic acid (**Figure 3A**) indicates that aconitase was dysfunctional. *Cis-*aconitate is an unexpected metabolite as it is only an intermediate in the isomerization of citrate to isocitrate. Given that aconitase has a [4Fe-4S] cluster in its active site, which is sensitive to decomposition by oxidative attack it is likely that ROS affected the function of aconitase, a phenomenon which has previously been observed in *P. fluorescens* (Middaugh et al., 2005). Other metals have been found to cause similar stress reactions in *Pseudomonas*. Exposure to high concentrations of zinc caused a shift in ATP production from oxidative to substrate-level phosphorylation and a simultaneous decrease of NADH and increase of NADPH production (Alhasawi et al., 2014). Vanadium toxicity was also linked to the Kreb’s cycle as mutations to the *idh* (coding for isocitrate dehydrogenase) and *acnD* (coding for an aconitase) genes increased resistance to this metal, presumably due to a change in expression to less metal-sensitive isozymes (Denayer et al., 2006). Overall our results indicate that the metabolic changes in *P. pseudoalcaligenes* KF707 were similar to those observed in *P. fluorescens*, but more pronounced when growing on Bp indicating that growth on this carbon source exacerbates stress caused by Al.

*O*-acetylserine is an intermediate in cysteine biosynthesis, and was increased with Bp but decreased with Sc (**Figure 3B**). Apart from being the assimilation point of inorganic sulfur and being used to synthesize all other sulfur-containing metabolites, cysteine is the amino acid that allows for the formation of disulfide bonds and iron-sulfur clusters in proteins as well as serving as an intermediate in the biosynthesis of glutathione, the main antioxidant within the cell (Brosnan and Brosnan, 2006). While cysteine could not be quantified, the only role of *o*-acetylserine is in the biosynthesis of cysteine. Cysteine, methionine, and sulfur metabolism were implicated by mBROLE (**Table 2**) in both carbon sources, implying that Al toxicity caused alterations to anti-oxidant production pathways.

All three polyamines that were detected, spermidine, putrescine, and cadaverine, have been implicated in oxidative stress resistance in *Escherichia coli* as they are able to scavenge free radicals (Chattopadhyay et al., 2003; Wortham et al., 2007). All of these compounds were decreased under Al exposure, cadaverine being specific to Sc and putrescine specific to Bp (**Figure 3B**, light purple symbols). Al induced oxidative stress could be expected to cause polyamine levels to increase, but since their free levels are normally very low as most cellular polyamines are complexed with nucleic acids (Wortham et al., 2007) an increase in ROS would rapidly deplete these free levels. Polyamines also induced the expression of acid resistance genes in *E. coli* that resulted in the secretion of 4-aminobutyric acid (Chattopadhyay and Tabor, 2013), which was secreted more in response to Al stress in Bp-grown cultures (Supplementary Figure S6A). In *E. coli* this secretion was due to the action of a glutamate/4-aminobutyric acid antiporter, of which *P. pseudoalcaligenes* KF707 does not possess a homolo based on a BLAST search (Triscari-Barberi et al., 2012). These cultures experienced a decrease in pH due to benzoic acid production, as well as a decrease in intracellular glutamate, but as the growth medium did not contain exogenous glutamate a system comparable to *E. coli* would not have functioned anyway. *P. pseudoalcaligenes* could still have been using this secreted 4-aminobutyric acid as a proton sink similarly to *E. coli* (Chattopadhyay et al., 2003). In Sc-grown cells exposed to Al, secretion of spermidine increased (Supplementary Figure S6). Spermidine was found to be associated with the outer membrane of *P. aeruginosa* and protected against exogenous oxidative stress; though the role of secreted spermidine was less clear (Johnson et al., 2012). In the present study the spermidine accumulated in the spent medium was likely derived from spermidine being exported for such a purpose. Intracellular putrescine has also been associated with increasing resistance to oxidative stress in *Burkholderia cenocepacia* (El-Halfawy and Valvano, 2014). Putrescine was decreased intracellularly in Bp-grown cells exposed to Al (**Figure 3B**) implicating it as an additional anti-oxidant polyamine. Polyamines were also implicated in resistance to oxidative stress induced by chromium in a number of environmental isolates (Joutey et al., 2014). Ornithine was decreased in Al exposed cells grown on both carbon sources (**Figure 3B**). Decreased levels of this amino acid precursor to putrescine (which is the precursor to spermidine) and the above further supports the use of polyamines to assist with resistance to oxidative stress caused by Al in *P. pseudoalcaligenes* KF707.

### Toxicity Effects of Copper

Metabolic profiles indicated that Cu exerted its toxicity through some different mechanisms than Al. Surprisingly, Cu appeared to cause some metabolic changes similar to Al that indicated that Cu was exerting oxidative stress, despite this possibility being definitively ruled out in *E. coli* (Macomber et al., 2007). As with Al, Cu induced the accumulation of glycolic acid and phosphoglycolic acid in Bp-grown cultures (**Figure 3C**) and increased secretion of glycolic acid in Sc-grown cultures (Supplementary Figure S6). While Cu alone may not cause oxidative stress, growth on Bp has the potential to generate far more ROS than Sc as complete catabolism of Bp requires four dioxygenases that use O_2_ to activate the conjugated carbons (Furukawa and Fujihara, 2008). In *E. coli* Cu was no more toxic and prevented toxicity when exogenous hydrogen peroxide was added (Macomber et al., 2007). In *P. pseudoalcaligenes* KF707, phosphoglycolic acid was accumulated indicating that the Cu added was not preventing oxidative stress but rather contributing to it. Transcriptional profiling of *P. aeruginosa* exposed to Cu during log phase showed changes to gene expression that were indicative of oxidative stress, which did not occur in cultures grown in the presence of Cu (Teitzel et al., 2006). Genes for active eﬄux of Cu were a main component of the response to Cu stress in both cultures indicating a non-metabolic response, especially compared to the elaborate metabolic re-configuring observed in *P. fluorescens* responding to Al (Mailloux et al., 2011). While oxidative stress was not implicated in gene expression profiles of *P. aeruginosa* grown in the presence of Cu, the observed buildup of glycolic and phosphoglycolic acid in *P. pseudoalcaligenes* KF707 indicate that ROS were present in Bp-grown cultures and were causing the same DNA damage discussed in the Al treated cultures. Oxalic acid was accumulated intracellularly with Bp (**Figure 3C**) and extracellarly with Sc (Supplementary Figure S6). This indicates that oxidative stress was occurring under Cu exposure as the glyoxylate/oxalate shunt induced in *P. fluorescens* under oxidative stress causes oxalic acid accumulation (Singh et al., 2009). Changes to polyamines were also observed in Cu exposed cultures. Spermidine secretion was again increased under Cu stress in Sc grown cultures (Supplementary Figure S6), however, intracellular levels increased with Bp (**Figure 3D**, purple symbols). Putrescine was not affected in either carbon source and instead cadaverine was decreased with Bp, indicating a possible shift in polyamine use for mitigating oxidative stress. The secretion of spermidine in Sc-grown cultures is of further interest as Cu surfaces have been demonstrated to kill bacteria via ROS mediated lipid peroxidation resulting in membrane destruction (Hong et al., 2012; Warnes et al., 2012). The polyamine pre-cursor ornithine was decreased only with Sc, thus together with the other observed changes this indicates that polyamines may not play as an important role in Bp-grown cultures under Cu stress compared to Al.

In *E. coli* Cu disrupts Fe–S clusters of dehydratases, such as those involved in the synthesis of branched chain amino acids (Macomber and Imlay, 2009). Here we observed that metabolites associated with this pathway, citramalic and isopropylmalic acid (**Figure 3C**, orange symbols) were affected by Cu, as well as the end-products valine, leucine, and isoleucine (**Figure 3D**, blue symbols) though not in an expected manner. In Sc-grown cultures valine was decreased while with Bp leucine was decreased but isoleucine increased. Isoleucine is synthesized by converting aspartate to threonine, which is then deaminated to make 2-oxobutanoate (Ogata et al., 1999). Citramalic acid was decreased in Bp grown cultures but was increased with Sc, as well as 2-isopropylmalic acid which is used to synthesize leucine. 2-Isopropylmalic acid is synthesized from 2-oxoisovalerate through a dehydratase-mediated reaction, which is susceptible to inhibition by Cu (Macomber and Imlay, 2009). This enzyme, dihydroxy acid-dehydratase is also used in isoleucine synthesis. As there was no consistent response to Cu stress (i.e., depletion of end-products and buildup of intermediates) no general conclusion can be made about the effect of Cu on branched-chain amino acid synthesis of *P. pseudoalcaligenes* grown on either carbon source. As these metabolites were definitely affected by Cu, quantification of enzyme activity and/or gene expression would help elucidate how Cu affected this pathway. Based on KEGG annotation (and BLAST searches), citramalic acid (both the R and S enantiomers) appears to be a dead-end metabolite in *P. pseudoalcaligenes* KF707 and cannot be synthesized from pyruvate and acetyl-CoA as in methanogens. This poses the question of why this metabolite was present in any cultures as well as why its amounts were affected by metal presence. It may have some uncharacterized role in metabolism when *Pseudomonas* sp. are grown on minimal media and so warrants further investigation, especially given *P. fluorescens’* tendency to alter its Kreb’s cycle under when stressed (Mailloux et al., 2011).

Fumarase, which converts fumarate to malate during normal functioning of the Krebs cycle, was also found to be inhibited by Cu toxicity in *E. coli* (Macomber and Imlay, 2009). In Bp-grown cultures exposed to Cu fumaric acid levels increased while malic acid decreased (**Figure 3C**, light blue symbols), indicating a similar disruption to fumarase occured in *P. pseudoalcaligenes* KF707. In *P. fluorescens* under Al or gallium stress expression of FumA/B isozymes was decreased while FumC increased as this isozyme does not require the use of an Fe-S cluster (Chenier et al., 2008). Based on BLAST searches of the KF707 genome, it does not possess a *fumC* gene and so would be unable overcome any inhibition to FumA/B (Altschul et al., 1990). Interestingly, *E. coli* grown on Sc was more susceptible to Cu toxicity acting through the inhibition of fumarase than when it was grown on glucose (Macomber and Imlay, 2009). Here it appears that *P. pseudoalcaligenes* KF707 is less susceptible when grown on Sc than when grown on Bp. Together these results emphasize the importance of considering how a bacterium is assimilating carbon for determining the effects of metal toxicity, a notion that is highly relevant for developing solutions for the bioremediation of co-contaminated sites.

Changes to various metabolites with possible roles as intracellular chelators were observed in Cu exposed cultures. Under Cu stress, Bp-grown cultures accumulated glycerol-3-phosphate, pyrophosphate and glycerophosphoglycerol (**Figure 3C**, green symbols). This metabolite’s secretion was also decreased while in Sc-grown cultures only glycerol-3-phosphate was accumulated and secretion of pyrophosphate decreased (Supplementary Figure S6). As phosphate was in excess due to its use as the buffering agent in the medium, it could not be quantified along with the other metabolites. In *E. coli* addition of Cu induced intracellular poly-phosphate degradation and export of phosphate (Grillo-Puertas et al., 2014). Polyphosphate has also been implicated in resistance to oxidative stress (Gray and Jakob, 2015). Thus combined stress from Cu and ROS generated by growing on Bp would likely create conflicting signals for poly-P accumulation or degradation whereas Sc-grown cells would only have incentive to degrade poly-P. This dichotomy could explain why only one phosphate containing metabolite, glycerophosphoglycerol, changed in the same fashion in Sc and Bp grown cells. Given the observed changes in phosphate containing metabolites further investigation into the possible role of poly-P in mitigating the combined toxicity of metals and organic pollutant catabolism is warranted, especially given the role phosphate can play in metal speciation in soils (Shahid et al., 2013).

Non-phosphate containing metabolites were also affected. Methionine was found to be a key intracellular chelator of Cu(I) in *E. coli*; when it was not present in the growth medium under anaerobic conditions free Cu(I) accumulated in the cytoplasm and interfered with Fe–S cluster assembly proteins (Fung et al., 2013). Here, methionine was increased in Bp grown cultures but decreased with Sc potentially indicating its similar use as an intracellular chelator (**Figure 3D**, blue symbol). Citric acid was also accumulated in the spent media of Cu exposed cultures grown on Bp (Supplementary Figure S6), a phenomenon that was previously observed to occur in *P. putida* grown on glucose but not aromatic substrates (Basu et al., 2009). Interestingly, this accumulation was only extracellular and was not observed in Al exposed cultures where it would have been a logical response as citrate chelates Al (Hue et al., 1986). Oxalate also strongly chelates Al and is generated under Al exposure by *P. fluorescens* (Singh et al., 2009). Here the secretion of this metabolite was increased by both metals in Sc-grown cultures, indicating its potential role as a general metal scavenger in less-stressed cells (Supplementary Figure S6). Glutamate has been observed to be accumulated as an osmoprotectant in *P. aeruginosa* (Behrends et al., 2010). In Bp-grown, Cu exposed cultures glutamate was increased and its secretion was decreased (**Figure 3D**, Supplementary Figure S6B), indicating that Cu toxicity elicited a response similar to osmotic stress. These results thus indicate that Cu toxicity causes changes to the secretion of multiple metabolites, an interesting result given that in *P. aeruginosa* Cu exposure induced many genes involved in its eﬄux (Teitzel et al., 2006). These observations, and the aforementioned Cu surface mediated lipid peroxidation (Hong et al., 2012; Warnes et al., 2012) indicate that further investigations into Cu toxicity should consider its interactions with both the inner and outer membrane and associated proteins.

β-alanine and alanine/aspartate/glutamate metabolism were implicated by pathway enrichment analysis in Cu exposed cultures grown on either carbon source (**Table 2**). Compared to other 4-carbon molecules being used as the sole carbon source, aspartate was previously observed to greatly increase the tolerance of *P. fluorescens* to Cu (Booth et al., 2013a). Aspartate and β-alanine can be interconverted and are basal intermediates in many biosynthetic pathways including pantothenate and CoA biosynthesis (Ogata et al., 1999), which were affected in Cu exposed cultures though they were just over the *p*-value cutoff (**Table 2**). Acetyl-CoA synthetase, which catalyzes the key reaction of adding the acetyl group to coenzyme A was found to be inhibited by Cu in wastewater treatment bacteria (Tsai et al., 2013). β-alanine was increased in both carbon sources in response to Cu exposure and asparate was increased in Bp grown cells. This could be an indication of a similar inhibition of acetyl-CoA synthetase by Cu occurring in *P. pseudoalcaligenes* KF707 and occurring more drastically when grown on Bp resulting in the additional accumulation of aspartate. Both β-alanine and acetyl-CoA synthesis were implicated in toxic effects of phenanthrene in *Sinorhizobium* (Keum et al., 2008) indicating possibly that Cu was exacerbating the toxicity of Bp. Inhibition of acetyl-CoA synthetase would be devastating to the cell and may explain our previous observation that Cu tolerance was lower in Bp-grown cultures than those grown on Sc (Booth et al., 2013a).

The results presented here have indicated that Cu toxicity elicits multiple metabolic changes that are suggestive of oxidative stress, a phenomenon that was comprehensively ruled out in *E. coli* (Macomber et al., 2007). These *E. coli* cultures were grown in rich or amino acid containing medium and were resistant to millimolar levels of Cu compared to the 60 μM used here. Our past work showed that the tolerance of *P. pseudoalcaligenes* and *P. fluorescens* to Cu decreased 100-fold from LB medium to MSM, which was used in the present study (Booth et al., 2013a). Contrary to *E. coli*, when the plant pathogen *Xanthomonas campestris* was exposed to Cu it became more susceptible to hydrogen peroxide and also upregulated ROS-detoxification genes (Sornchuer et al., 2014). Thus it seems possible that Cu can exert toxicity through ROS-mediated mechanisms under particular conditions in some organisms.

### Effects of Metal Toxicity on Biphenyl Metabolism

**FIGURE 4:**
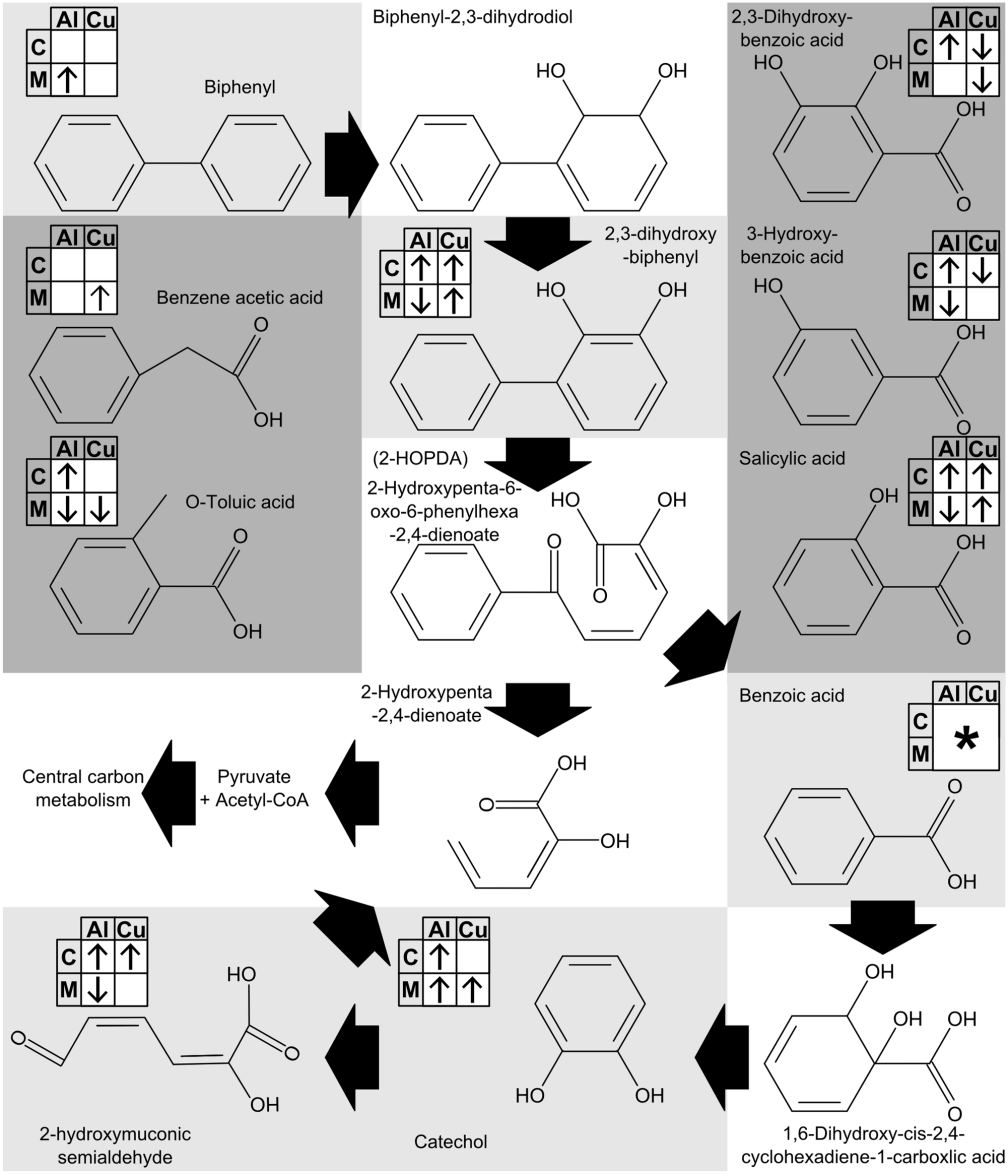
**Changes to metabolites derived from Bp degradation in cells (C) and spent media (M) of cultures of *P. pseudoalcaligenes* KF707 grown on Bp as the sole carbon source and exposed to Al or Cu.** Arrows indicate whether a metabolite was increased (↑) or decreased (↓) in response to each metal. Values were derived from VIP and *p*(corr) from OPLS-DA models comparing control to metal exposed samples. Empty boxes mean there was no significant change (VIP < 0.8). For details, see Supplementary Figure S6. Benzoic acid was detected but could not be accurately quantitated due to elevated concentrations resulting in detector saturation (^∗^). Metabolite background shading denotes whether it is a cannonical Bp degradation product and was detected (light gray) or not detected (white). Several metabolites that are not involved in Bp degradation but are structurally related were also detected (dark gray).

Under exposure to both metals changes to Bp-degradation intermediates were observed, as well as changes to several structurally related metabolites (Supplementary Figure S7, summarized in **Figure 4**). Also, benzoate degradation was implicated by mBROLE with both metals (**Table 2**). Bp itself was only changed in the spent media from Al exposed cultures, and was increased. Bp is normally insoluble in water, so this is of interest as it could indicate that bacterial activity, potentially the decrease in pH, increased solubility. No metabolites with obvious surfactant properties were identified, though they could be present within the unknown compounds. This warrants further investigation as increasing the solubility of pollutants by biosurfactants has been found to improve degradation (Cameotra et al., 2010). In *P. pseudoalcaligenes* KF707 Bp metabolism begins with dioxygenation to produce 2,3-dihydroxybiphenyl (as originally characterized by Furukawa and Miyazaki, 1986), which was increased in Al exposed cells but decreased in the media and increased in both sample types with Cu (**Figure 4**). These increases indicate inhibition of the next step of Bp metabolism, ring opening by 2,3-dihydroxybiphenyl 1,2-dioxygenase. Both Bp and 2,3-dihydroxy biphenyl dioxygenase, are heteromultimeric Rieske-type non-heme oxygenases that contain catalytically active [2Fe–2S] clusters (Gibson and Parales, 2000). As was previously noted, both Al and Cu have been characterized as damaging iron–sulfur clusters, and thus these results indicate that the iron–sulfur clusters of aromatic oxygenases are likely targets of metal toxicity. After 2,3-dihydroxybiphenyl is cleaved to produce benzoic acid and 2-hydroxy-2,4-pentadienoate the benzoic acid is dioxygenated and decarboxylated to catechol, which was accumulated under all conditions except Cu exposed cells (**Figure 4**). Benzoic acid was also accumulated to an unquantifiable, GC-MS detector saturating level in all samples. This indicates that both benzoic acid 1,2-dioxygenase and catechol oxygenase may have been inhibited by both metals. Catechol can be processed either into the β-keto-adipate pathway (starting with catechol-1,2-dioxygenase) or the pyruvate/acetyl-CoA pathway (starting with catechol-2,3-dioxygenase). From genomic characterization, both pathways appear to be present in *P. pseudoalcaligenes* KF707 (Triscari-Barberi et al., 2012). An unknown analyte was identified as 2-hydroxymuconic semialdehyde (see Supplementary Material) indicating the use of catechol-2,3-dioxygenase, which was confirmed spectroscopically using a cell-free enzymatic assay (results not shown). Unexpectedly, given the accumulation of the upstream metabolites, 2-hydroxymuconic semialdehyde was also accumulated, though only in cells, it was decreased in the spent media of Al exposed samples. As all intermediates detected were increased by both metals, it could be surmised that the stress induced created a greater demand for carbon and energy, resulting in greater quantities of all intermediates. The alternative hypothesis of iron–sulfur clusters of aromatic dioxygenase being damaged by two different metals, which would mediate this damage in very different ways, makes for an intriguingly broad explanation for why metals inhibit organic pollutant catabolism. Identification of the exact mechanisms by which Al and Cu, as well as other metals, inhibit aromatic oxygenases would enable the development of possible ways to prevent this inhibition and improve bioremediation of co-contaminated sites.

Several unexpected metabolites similar to benzoic acid were identified and quantified: salicylic, 3-hydroxybenzoic, 2,3-dihydroxybenzoic, benzeneacetic and *O*-toluic acid (**Figure 4**). While these metabolites have been found in other biological systems, they are not intermediates of Bp degradation. Their presence here implies possible non-specific action of oxygenases from Bp catabolism or reactions between Bp degradation intermediates and ROS. The two hydroxybenzoic acids that were observed could have been formed by OH^∙^ reaction with benzoic acid, which is favored at the 2-position (Tanaka, 2013) and subsequent deprotonation and ring closure. 2,3-dihydroxybenzoic acid could have been formed by an additional such reaction. Alternatively, 2,3-dihydroxybenzoic acid may have been formed by catechol 2,3-dioxygenase in the presence of excess benzoic acid substrate from Bp cleavage. A final possible explanation for this compound’s presence is its intentional production for use in siderophores (O’Brien et al., 1970) for iron acquisition or as a protective measure from the metals that were added. Phenylacetic and *O*-toluic acid are the most unexpected metabolites as these cannot be formed directly from ROS interactions with Bp degradation intermediates and so were most likely derived from incorrect degradation of Bp. All of these unexpected metabolites were altered by the presence of metals, indicating that their formation had some relation to Al and Cu. As these metals appeared to be inhibiting the aromatic oxygenases, it could be possible that the interaction between the metals and the oxygenases was causing non-specific reactions to occur. The generation of dead-end metabolites due to metals thus presents a possible mechanism of enhanced toxicity.

### Metabolic Changes and Implication of Oxidative Stress

The metabolic changes caused by Al and Cu in cultures grown on Bp repeatedly implied that oxidative stress was increased compared to Sc grown cultures. Many other studies have found that organic pollutants cause oxidative stress in bacteria. 1,3-dichloroprop-1-ene induced oxidative stress during its degradation in *P. pavonaceae* (Nikel et al., 2013) which resulted in the accumulation of the antioxidant NADPH, a metabolite who’s production was implicated in oxidative stress by others (Mailloux et al., 2011). In *Pseudomonas* sp. strain As1 overexpression of ROS detoxifying enzymes enhanced the degradation of naphthalene (Kang et al., 2007). In *B. xenovorans* LB400 growth on Bp induced expression of alkyl hydroperoxide reductase, which is also expressed under hydrogen peroxide treatment (Agulló et al., 2007). Supplementation of this strain with the antioxidant α–tocopherol decreased the amount of time needed to degrade poly-chlorinated Bps in soil (Ponce et al., 2011). In another *Pseudomonas* sp. (strain B4) growth on 2-chlorobiphenyl induced ROS generation (Chávez et al., 2004). Other metabolomic studies have also found evidence for ROS-based toxicity: the pesticide 2,4-dichlorophenoxyacetic acid (2,4-D) was found to cause oxidative stress in *E. coli* and *Rhizobium leguminosarum* (Bhat et al., 2015, 2014), as well as nicotine in a *Pseudomonas* species (Ye et al., 2012) and phenanthrene in *Sinorhizobium* (Keum et al., 2008). This phenomenon is not restricted to Gram negative proteobacteria; when *Rhodococcus aetherivorans* I24 was grown in polychlorinated biphenyl contaminated soil microarray analysis showed that the expression of many stress related genes such as chaperones and oxidative stress protection genes were increased (Puglisi et al., 2010). As complete catabolism of Bp by *P. pseudoalcaligenes* KF707 requires four O_2_-using dioxygenases (Furukawa and Miyazaki, 1986; Taira et al., 1992), aerobic metabolism causes the generation of ROS in all organisms (Asad et al., 2004), and following from the above studies, oxidative stress caused by Bp metabolism is thus the most likely explanation for the observed metabolic differences caused by Al and Cu.

## Conclusion

Succinate and Bp are assimilated into central metabolism completely differently, with Sc as a component of the Krebs cycle whereas complete catabolism of Bp requires 10 catabolic enzymes and six intermediate steps to enter central metabolism. Here we have shown that these differences in assimilation cause the toxicity of Al and Cu to be exacerbated when Bp is the carbon source. This was manifested as changes to more metabolites involved in a wider variety of functions than those affected in cultures grown on Sc. As the concentrations of metals were the same for both carbon sources, we have thus demonstrated that metal toxicity is physiologically more pronounced when bacteria are using a complex aromatic substrate as their sole carbon source. Though the affected metabolites were diverse in their role within the cell, they are united by their implication in oxidative stress from studies in other systems. This makes the understanding how organic pollutants induce oxidative stress, especially in conjunction with metals, a topic of paramount importance for further laboratory and applied studies on the problem environmental co-contamination.

## Author Contributions

SB designed and carried out experiments, analyzed data and wrote manuscript. AW and RT contributed to experimental design, data interpretation and manuscript preparation.

## Conflict of Interest Statement

The authors declare that the research was conducted in the absence of any commercial or financial relationships that could be construed as a potential conflict of interest.

## Abbreviations

KEGG: Kyoto encyclopedia of genes and genomes
OPLS-DA: orthogonal partial least squares discriminant analysis
PCA: principal component analysis
PPP: pentose-phosphate pathway
ROS: reactive oxygen species
VIP: variable influence on projection

## References

[B1] Agulló L. Cámara B. Martínez P. Latorre V. Seeger M. (2007). Response to (chloro)biphenyls of the polychlorobiphenyl-degrader *Burkholderia xenovorans* LB400 involves stress proteins also induced by heat shock and oxidative stress. *FEMS Microbiol. Lett.* 267 167–175. 10.1111/j.1574-6968.2006.00554.x 17166226

[B2] Alberts B. Johnson A. Lewis J. Raff M. Roberts K. Walter P. (2002). “DNA Repair,” in *Molecular Biology of the Cell* (New York: Garland Science). Available at: http://www.ncbi.nlm.nih.gov/books/NBK26879/ (accessed January 26, 2015).

[B3] Alhasawi A. Auger C. Appanna V. P. Chahma M. Appanna V. D. (2014). Zinc toxicity and ATP production in *Pseudomonas fluorescens*. *J. Appl. Microbiol.* 117 65–73. 10.1111/jam.12497 24629129

[B4] Allen E. W. (2008). Process water treatment in Canada’s oil sands industry: I. Target pollutants and treatment objectives. *J. Environ. Eng. Sci.* 7 123–138. 10.1139/S07-038

[B5] Altschul S. F. Gish W. Miller W. Myers E. W. Lipman D. J. (1990). Basic local alignment search tool. *J. Mol. Biol.* 215 403–410. 10.1016/S0022-2836(05)80360-2 2231712

[B6] Annicchiarico C. Buonocore M. Cardellicchio N. Di Leo A. Giandomenico S. Spada L. (2011). PCBs, PAHs and metal contamination and quality index in marine sediments of the Taranto Gulf. *Chem. Ecol.* 27 21–32. 10.1080/02757540.2010.536156

[B7] Asad N. R. Asad L. M. B. O. de Almeida C. E. B. Felzenszwalb I. Cabral-Neto J. B. Leitão A. C. (2004). Several pathways of hydrogen peroxide action that damage the *E. coli* genome. *Genet. Mol. Biol.* 27 291–303. 10.1590/S1415-47572004000200026

[B8] Basu A. Das D. Bapat P. Wangikar P. P. Phale P. S. (2009). Sequential utilization of substrates by *Pseudomonas putida* CSV86: signatures of intermediate metabolites and online measurements. *Microbiol. Res.* 164 429–437. 10.1016/j.micres.2007.02.008 17467253

[B9] Behrends V. Ryall B. Wang X. Bundy J. G. Williams H. D. (2010). Metabolic profiling of *Pseudomonas aeruginosa* demonstrates that the anti-sigma factor MucA modulates osmotic stress tolerance. *Mol. Biosyst.* 6 562–569. 10.1039/b918710c 20174684

[B10] Berthon G. (2002). Aluminium speciation in relation to aluminium bioavailability, metabolism and toxicity. *Coord. Chem. Rev.* 228 319–341. 10.1016/S0010-8545(02)00021-8

[B11] Bhat S. V Booth S. C. McGrath S. G. K. Dahms T. E. S. (2014). *Rhizobium leguminosarum* bv. viciae 3841 adapts to 2,4-dichlorophenoxyacetic acid with “Auxin-Like” morphological changes, cell envelope remodeling and upregulation of central metabolic pathways. *PLoS ONE* 10:e0123813 10.1371/journal.pone.0123813 PMC4412571 25919284

[B12] Bhat S. V Booth S. C. Vantomme E. A. Afroj S. Yost C. K. Dahms T. E. (2015). Oxidative stress and metabolic perturbations in *Escherichia coli* exposed to sublethal levels of 2,4-dichlorophenoxyacetic acid. *Chemosphere* 135 453–461. 10.1016/j.chemosphere.2014.12.035 25661029

[B13] Booth S. C. George I. F. S. Zannoni D. Cappelletti M. Duggan G. E. Ceri H. (2013a). Effect of aluminium and copper on biofilm development of *Pseudomonas pseudoalcaligenes* KF707 and P. fluorescens as a function of different media compositions. *Metallomics* 5 723–735. 10.1039/c3mt20240b 23604327

[B14] Booth S. C. Weljie A. M. Turner R. J. (2013b). Computational tools for the secondary analysis of metabolomics experiments. *Comput. Struct. Biotechnol. J.* 4 e201301003 10.5936/csbj.201301003 PMC3962093 24688685

[B15] Booth S. C. Workentine M. L. Weljie A. M. Turner R. J. (2011a). Metabolomics and its application to studying metal toxicity. *Metallomics* 3 1142 10.1039/c1mt00070e 21922109

[B16] Booth S. C. Workentine M. L. Wen J. Shaykhutdinov R. Vogel H. J. Ceri H. (2011b). Differences in metabolism between the biofilm and planktonic response to metal stress. *J. Proteome Res.* 10 3190–3199. 10.1021/pr2002353 21561166

[B17] Broeckling C. D. Reddy I. R. Duran A. L. Zhao X. Sumner L. W. Division P. B. (2006). MET-IDEA: data extraction tool for mass spectrometry-based metabolomics. *Anal. Chem.* 78 4334–4341. 10.1021/ac0521596 16808440

[B18] Brosnan J. T. Brosnan M. E. (2006). The sulfur-containing amino acids: an overview. *J. Nutr.* 136(6 Suppl.) 1636S–1640S. 16702333 10.1093/jn/136.6.1636S

[B19] Burgess R. M. Terletskaya A. V. Milyukin M. V. Povolotskii M. Demchenko V. Y. Bogoslavskaya T. A. (2009). Concentration and distribution of hydrophobic organic contaminants and metals in the estuaries of Ukraine. *Mar. Pollut. Bull.* 58 1103–1115. 10.1016/j.marpolbul.2009.04.013 19446854

[B20] Cameotra S. S. Makkar R. S. Kaur J. Mehta S. K. (2010). “Synthesis of biosurfactants and their advanatages to microorganisms and mankind,” in *Biosurfactants Advances in Experimental Medicine and Biology* ed. Sen R. (New York, NY: Springer) 261–280. 10.1007/978-1-4419-5979-9 20545289

[B21] Chagoyen M. Pazos F. (2011). MBRole: enrichment analysis of metabolomic data. *Bioinformatics* 27 730–731. 10.1093/bioinformatics/btr001 21208985

[B22] Chattopadhyay M. K. Tabor C. W. Tabor H. (2003). Polyamines protect *Escherichia coli* cells from the toxic effect of oxygen. *Proc. Natl. Acad. Sci. U.S.A.* 100 2261–2265. 10.1073/pnas.2627990100 12591940 PMC151328

[B23] Chattopadhyay M. K. Tabor H. (2013). Polyamines are critical for the induction of the glutamate decarboxylase-dependent acid resistance system in *Escherichia coli*. *J. Biol. Chem.* 288 33559–33570. 10.1074/jbc.M113.510552 24097985 PMC3837104

[B24] Chávez F. P. Lünsdorf H. Jerez C. A. (2004). Growth of polychlorinated-biphenyl-degrading bacteria in the presence of biphenyl and chlorobiphenyls generates oxidative stress and massive accumulation of inorganic polyphosphate. *Appl. Environ. Microbiol.* 70 3064–3072. 10.1128/AEM.70.5.3064-3072.2004 15128568 PMC404396

[B25] Chenier D. Beriault R. Mailloux R. Baquie M. Abramia G. Lemire J. (2008). Involvement of fumarase C and NADH oxidase in metabolic adaptation of *Pseudomonas fluorescens* cells evoked by aluminum and gallium toxicity. *Appl. Environ. Microbiol.* 74 3977–3984. 10.1128/AEM.02702-07 18469122 PMC2446511

[B26] Darnault C. Rockne K. Stevens A. Mansoori G. A. Sturchio N. (2005). Fate of environmental pollutants. *Water Environ. Res.* 77 2576–2658. 10.2175/106143005x54632

[B27] Denayer S. Matthijs S. Cornelis P. (2006). Resistance to vanadium in *Pseudomonas fluorescens* ATCC 17400 caused by mutations in TCA cycle enzymes. *FEMS Microbiol. Lett.* 264 59–64. 10.1111/j.1574-6968.2006.00435.x 17020548

[B28] Dieterle F. Ross A. Schlotterbeck G. Senn H. (2006). Probabilistic quotient normalization as a robust method to account for dilution of complex biological mixtures. Application in 1H NMR metabonomics. *Anal. Chem.* 78 4281–4290. 10.1021/ac051632c 16808434

[B29] El-Halfawy O. M. Valvano M. A. (2014). Putrescine reduces antibiotic-induced oxidative stress as a mechanism of modulation of antibiotic resistance in *Burkholderia cenocepacia*. *Antimicrob. Agents Chemother.* 58 4162–4171. 10.1128/AAC.02649-14 24820075 PMC4068564

[B30] Exley C. (2004). The pro-oxidant activity of aluminum. *Free Radic. Biol. Med.* 36 380–387. 10.1016/j.freeradbiomed.2003.11.017 15036357

[B31] Exley C. (2009). Darwin, natural selection and the biological essentiality of aluminium and silicon. *Trends Biochem. Sci.* 34 589–593. 10.1016/j.tibs.2009.07.006 19773172

[B32] Ferreira P. A. A. Bomfeti C. A. Soares B. L. de Souza Moreira F. M. (2012). Efficient nitrogen-fixing *Rhizobium* strains isolated from amazonian soils are highly tolerant to acidity and aluminium. *World J. Microbiol. Biotechnol.* 28 1947–1959. 10.1007/s11274-011-0997-7 22806016

[B33] Fornalczyk A. Willner J. Francuz K. Cebulski J. (2013). E-waste as a source of valuable metals. *Arch. Mater. Sci. Eng.* 63 87–92.

[B34] Fung D. K. C. Lau W. Y. Chan W. T. Yan A. (2013). Copper eﬄux is induced during anaerobic amino acid limitation in *Escherichia coli* to protect iron-sulfur cluster enzymes and biogenesis. *J. Bacteriol.* 195 4556–4568. 10.1128/JB.00543-13 23893112 PMC3807430

[B35] Furukawa K. Fujihara H. (2008). Microbial degradation of polychlorinated biphenyls: biochemical and molecular features. *J. Biosci. Bioeng.* 105 433–449. 10.1263/jbb.105.433 18558332

[B36] Furukawa K. Miyazaki T. (1986). Cloning of a gene cluster encoding biphenyl and chlorobiphenyl degradation in *Pseudomonas pseudoalcaligenes*. *J. Bacteriol.* 166 392–398. 3009395 10.1128/jb.166.2.392-398.1986 PMC214617

[B37] Giandomenico A. R. Cerniglia G. E. Biaglow J. E. Stevens C. W. Koch C. J. (1997). The importance of sodium pyruvate in assessing damage produced by hydrogen peroxide. *Free Radic. Biol. Med.* 23 426–434. 10.1016/S0891-5849(97)00113-5 9214579

[B38] Gibson D. T. Parales R. E. (2000). Aromatic hydrocarbon dioxygenases in environmental biotechnology. *Curr. Opin. Biotechnol.* 11 236–243. 10.1016/S0958-1669(00)00090-2 10851146

[B39] Gray M. J. Jakob U. (2015). Oxidative stress protection by polyphosphate-new roles for an old player. *Curr. Opin. Microbiol.* 24C 1–6. 10.1016/j.mib.2014.12.004 25589044 PMC4380828

[B40] Grillo-Puertas M. Schurig-Briccio L. A. Rodríguez-Montelongo L. Rintoul M. R. Rapisarda V. A. (2014). Copper tolerance mediated by polyphosphate degradation and low-affinity inorganic phosphate transport system in *Escherichia coli*. *BMC Microbiol.* 14:72 10.1186/1471-2180-14-72 PMC3994843 24645672

[B41] Gullberg J. Jonsson P. Nordström A. Sjöström M. Moritz T. (2004). Design of experiments: an efficient strategy to identify factors influencing extraction and derivatization of *Arabidopsis thaliana* samples in metabolomic studies with gas chromatography/mass spectrometry. *Anal. Biochem.* 331 283–295. 10.1016/j.ab.2004.04.037 15265734

[B42] Hassanvand M. S. Naddafi K. Faridi S. Nabizadeh R. Sowlat M. H. Momeniha F. (2015). Characterization of PAHs and metals in indoor/outdoor PM10/PM2.5/PM1 in a retirement home and a school dormitory. *Sci. Total Environ.* 527–528, 100-110. 10.1016/j.scitotenv.2015.05.001 25958359

[B43] Hong R. Kang T. Y. Michels C. A. Gadura N. (2012). Membrane lipid peroxidation in copper alloy-mediated contact killing of *Escherichia coli*. *Appl. Environ. Microbiol.* 78 1776–1784. 10.1128/AEM.07068-11 22247141 PMC3298164

[B44] Hue N. V. Craddock G. R. Adams F. (1986). Effect of organic acids on aluminum toxicity in subsoils. *Soil Sci. Soc. Am. J.* 50 28 10.2136/sssaj1986.03615995005000010006x

[B45] Hummel J. Selbig J. Walther D. Kopka J. (2007). “The Golm Metabolome Database: a database for GC-MS based metabolite profiling,” in *Topics in Current Genetics* eds Nielsen J. Jewett M. C. (Heidelberg: Springer-Verlag Berlin Heidelberg) 75–95. 10.1007/4735.

[B46] Imlay J. A. (2003). Pathways of oxidative damage. *Annu. Rev. Microbiol.* 57 395–418. 10.1146/annurev.micro.57.030502.090938 14527285

[B47] Itai T. Otsuka M. Asante K. A. Muto M. Opoku-Ankomah Y. Ansa-Asare O. D. (2014). Variation and distribution of metals and metalloids in soil/ash mixtures from Agbogbloshie e-waste recycling site in Accra, Ghana. *Sci. Total Environ.* 470–471, 707–716. 10.1016/j.scitotenv.2013.10.037 24184547

[B48] Jartun M. Pettersen A. (2010). Contaminants in urban runoff to Norwegian fjords. *J. Soils Sediments* 10 155–161. 10.1007/s11368-009-0181-y

[B49] Johnson L. Mulcahy H. Kanevets U. Shi Y. Lewenza S. (2012). Surface-localized spermidine protects the *Pseudomonas aeruginosa*: outer membrane from antibiotic treatment and oxidative stress. *J. Bacteriol.* 194 813–826. 10.1128/JB.05230-11 22155771 PMC3272965

[B50] Joutey N. T. Sayel H. Bahafid W. Ghachtouli N.E. (2014). Effet des polyamines sur la réduction du chrome hexavalent par des souches bactériennes et leur résistance. *Base* 18 4 Available at: http://popups.ulg.ac.be/1780-4507/index.php?id=11622 (accessed February 12, 2015).

[B51] Kang Y.-S. S. Lee Y. Jung H. Jeon C. O. Madsen E. L. Park W. (2007). Overexpressing antioxidant enzymes enhances naphthalene biodegradation in *Pseudomonas* sp. strain As1. *Microbiology* 153 3246–3254. 10.1099/mic.0.2007/008896-0 17906124

[B52] Keum Y. S. Seo J. S. Li Q. X. Kim J. H. (2008). Comparative metabolomic analysis of *Sinorhizobium* sp. C4 during the degradation of phenanthrene. *Appl. Microbiol. Biotechnol.* 80 863–872. 10.1007/s00253-008-1581-4 18668240 PMC7419452

[B53] Kuznetsova A A Knorre D. G. Fedorova O. S. (2009). Oxidation of DNA and its components with reactive oxygen species. *Russ. Chem. Rev.* 78 659–678. 10.1070/RC2009v078n07ABEH004038

[B54] Lemire J. A. Harrison J. J. Turner R. J. (2013). Antimicrobial activity of metals: mechanisms, molecular targets and applications. *Nat. Rev. Microbiol.* 11 371–384. 10.1038/nrmicro3028 23669886

[B55] Lemire J. Mailloux R. Auger C. Whalen D. Appanna V. D. (2010). *Pseudomonas fluorescens* orchestrates a fine metabolic-balancing act to counter aluminium toxicity. *Environ. Microbiol.* 12 1384–1390. 10.1111/j.1462-2920.2010.02200.x 20353438

[B56] Liu M. Huang B. Bi X. Ren Z. Sheng G. Fu J. (2013). Heavy metals and organic compounds contamination in soil from an e-waste region in South China. *Environ. Sci. Process. Impacts* 15 919–929. 10.1039/c3em00043e 23558980

[B57] Macdonald T. L. Bruce Martin R. (1988). Aluminum ion in biological systems. *Trends Biochem. Sci.* 13 15–19. 10.1016/0968-0004(88)90012-6 3072691

[B58] Macomber L. Imlay J. A (2009). The iron-sulfur clusters of dehydratases are primary intracellular targets of copper toxicity. *Proc. Natl. Acad. Sci. U.S.A.* 106 8344–8349. 10.1073/pnas.0812808106 19416816 PMC2688863

[B59] Macomber L. Rensing C. Imlay J. A. (2007). Intracellular copper does not catalyze the formation of oxidative DNA damage in *Escherichia coli*. *J. Bacteriol.* 189 1616–1626. 10.1128/JB.01357-06 17189367 PMC1855699

[B60] Mailloux R. J. Lemire J. Appanna V. D. (2011). Metabolic networks to combat oxidative stress in *Pseudomonas fluorescens*. *Antonie van Leeuwenhoek* 99 433–442. 10.1007/s10482-010-9538-x 21153706

[B61] Middaugh J. Hamel R. Jean-Baptiste G. Beriault R. Chenier D. Appanna V. D. (2005). Aluminum triggers decreased aconitase activity via Fe-S cluster disruption and the overexpression of isocitrate dehydrogenase and isocitrate lyase: a metabolic network mediating cellular survival. *J. Biol. Chem.* 280 3159–3165. 10.1074/jbc.M411979200 15548528

[B62] Mujika J. I. Rezabal E. Mercero J. M. Ruipérez F. Costa D. Ugalde J. M. (2014). Aluminium in biological environments: a computational approach. *Comput. Struct. Biotechnol. J.* 9:e201403002 10.5936/csbj.201403002 PMC3995234 24757505

[B63] Nikel P. I. Pérez-Pantoja D. de Lorenzo V. (2013). Why are chlorinated pollutants so difficult to degrade aerobically? Redox stress limits 1,3-dichloroprop-1-ene metabolism by *Pseudomonas pavonaceae*. *Philos. Trans. R. Soc. Lond. B. Biol. Sci.* 368 20120377 10.1098/rstb.2012.0377 PMC3638467 23479756

[B64] O’Brien I. G. Cox G. B. Gibson F. (1970). Biologically active compounds containing 2,3-dihydroxybenzoic acid and serine formed by *Escherichia coli*. *Biochim. Biophys. Acta* 201 453–460. 10.1016/0304-4165(70)90165-0. 4908639

[B65] Ogata H. Goto S. Sato K. Fujibuchi W. Bono H. Kanehisa M. (1999). KEGG: Kyoto encyclopedia of genes and genomes. *Nucleic Acids Res.* 27 29–34. 10.1093/nar/27.1.29 9847135 PMC148090

[B66] Olaniran A. O. Balgobind A. Pillay B. (2013). Bioavailability of heavy metals in soil: impact on microbial biodegradation of organic compounds and possible improvement strategies. *Int. J. Mol. Sci.* 14 10197–10228. 10.3390/ijms140510197 23676353 PMC3676836

[B67] Pellicer M. T. Aguilar J. Badia J. Baldoma L. (2003). Role of 2-phosphoglycolate phosphatase of *Escherichia coli* in metabolism of the 2-phosphoglycolate formed in DNA repair. *J. Bacteriol.* 185 5815–5821. 10.1128/JB.185.19.5815 13129953 PMC193966

[B68] Ponce B. L. Latorre V. K. González M. Seeger M. (2011). Antioxidant compounds improved PCB-degradation by *Burkholderia xenovorans* strain LB400. *Enzyme Microb. Technol.* 49 509–516. 10.1016/j.enzmictec.2011.04.021 22142725

[B69] Pradhan J. K. Kumar S. (2014). Informal e-waste recycling: environmental risk assessment of heavy metal contamination in Mandoli industrial area, Delhi, India. *Environ. Sci. Pollut. Res. Int.* 21 7913–7928. 10.1007/s11356-014-2713-2 24652574

[B70] Puglisi E. Cahill M. J. Lessard P. A. Capri E. Sinskey A. J. Archer J. A C. (2010). Transcriptional response of *Rhodococcus aetherivorans* i24 to polychlorinated biphenyl-contaminated sediments. *Microb. Ecol.* 60 505–515. 10.1007/s00248-010-9650-5 20369357

[B71] Renzi M. Perra G. Guerranti C. Mariottini M. Baroni D. Volterrani M. (2009). Assessment of environmental pollutants in ten southern Italy harbor sediments. *Toxicol. Ind. Health* 25 351–363. 10.1177/0748233709104868 19651808

[B72] Robinson B. H. (2009). E-waste: an assessment of global production and environmental impacts. *Sci. Total Environ.* 408 183–191. 10.1016/j.scitotenv.2009.09.044 19846207

[B73] Sandrin T. R. Chech A. M. Maier R. M. (2000). A rhamnolipid biosurfactant reduces cadmium toxicity during naphthalene biodegradation. *Appl. Environ. Microbiol.* 66 4585–4588. 10.1128/AEM.66.10.4585-4588.2000 11010924 PMC92350

[B74] Sandrin T. R. Hoffman D. R. (2007). “Bioremediation of organic and metal co-contaminated environments: effects of metal toxicity, speciation, and bioavailability on biodegradation,” in *Environmental Bioremediation Technologies* eds Singh S. Tripathi R. (Berlin: Springer) 1–34. 10.1007/978-3-540-34793-4_1

[B75] Shahid M. Xiong T. Masood N. Leveque T. Quenea K. Austruy A. (2013). Influence of plant species and phosphorus amendments on metal speciation and bioavailability in a smelter impacted soil: a case study of food-chain contamination. *J. Soils Sediments* 14 655–665. 10.1007/s11368-013-0745-8

[B76] Shively J. M. van Keulen G. Meijer W. G. (1998). Something from almost nothing: carbon dioxide fixation in chemoautotrophs. *Annu. Rev. Microbiol.* 52 191–230. 10.1146/annurev.micro.52.1.191 9891798

[B77] Shukla K. P. Singh N. K. Sharma S. (2010). Bioremediation: developments, current practices and perspectives. *Genet. Eng. Biotechnol. J.* 2010 1–20.

[B78] Silipo A. Molinaro A. (2010). The diversity of the core oligosaccharide in lipopolysaccharides. *Subcell. Biochem.* 53 69–99. 10.1007/978-90-481-9078-2_4 20593263

[B79] Singh R. Beriault R. Middaugh J. Hamel R. Chenier D. Appanna V. D. (2005). Aluminum-tolerant *Pseudomonas fluorescens*: ROS toxicity and enhanced NADPH production. *Extremophiles* 9 367–373. 10.1007/s00792-005-0450-7 15970995

[B80] Singh R. Lemire J. Mailloux R. J. Appanna V. D. (2008). A novel strategy involved anti-oxidative defense: the conversion of NADH into NADPH by a metabolic network. *PLoS ONE* 3:e2682 10.1371/journal.pone.0002682 PMC2443280 18628998

[B81] Singh R. Lemire J. Mailloux R. J. Chénier D. Hamel R. Appanna V. D. (2009). An ATP and oxalate generating variant tricarboxylic acid cycle counters aluminum toxicity in *Pseudomonas fluorescens*. *PLoS ONE* 4:e7344 10.1371/journal.pone.0007344 PMC2752808 19809498

[B82] Sornchuer P. Namchaiw P. Kerdwong J. Charoenlap N. Mongkolsuk S. Vattanaviboon P. (2014). Copper chloride induces antioxidant gene expression but reduces ability to mediate H2O2 toxicity in *Xanthomonas campestris*. *Microbiology* 160 458–466. 10.1099/mic.0.072470-0 24385479

[B83] Stein S. E. (1999). An integrated method for spectrum extractionand compound identification from gaschromatography/mass spectrometry data. *J. Am. Soc. Mass Spectrom.* 10 770–781. 10.1016/S1044-0305(99)00047-1

[B84] Stein S. E. (2011). *The NIST Mass Spectrometry Data Center 1A: NIST/EPA/NIH Mass Spectral Database (NIST 11) and NIST Mass Spectral Search Program (Version 2.0g).* Available at: http://www.nist.gov/srd/upload/NIST1a11Ver2-0Man.pdf

[B85] Stein S. E. Babushok V. I. Brown R. L. Linstrom P. J. (2007). Estimation of Kováts retention indices using group contributions. *J. Chem. Inf. Model.* 47 975–980. 10.1021/ci600548y 17367127

[B86] Stohs S. J. Bagchi D. (1995). Oxidative mechanisms in the toxicity of metal ions. *Free Radic. Biol. Med.* 18 321–336. 10.1016/0891-5849(94)00159-H 7744317

[B87] Taira K. Hirose J. Hayashida S. Furukawa K. (1992). Analysis of bph operon from the polychlorinated biphenyl-degrading strain of *Pseudomonas pseudoalcaligenes* KF707. *J. Biol. Chem.* 267 4844–4853. 1537863

[B88] Tanaka N. (2013). Density functional theory studies on the addition and abstraction reactions of OH radical with benzoate anion. *Open J. Phys. Chem.* 3 7–13. 10.4236/ojpc.2013.31002.

[B89] Teitzel G. M. Geddie A. De Long S. K. Kirisits M. J. Whiteley M. Parsek M. R. (2006). Survival and growth in the presence of elevated copper: transcriptional profiling of copper-stressed *Pseudomonas aeruginosa*. *J. Bacteriol.* 188 7242–7256. 10.1128/JB.00837-06 17015663 PMC1636237

[B90] Tremaroli V. Fedi S. Turner R. J. Ceri H. Zannoni D. (2008). *Pseudomonas pseudoalcaligenes* KF707 upon biofilm formation on a polystyrene surface acquire a strong antibiotic resistance with minor changes in their tolerance to metal cations and metalloid oxyanions. *Arch. Microbiol.* 190 29–39. 10.1007/s00203-008-0360-z 18437359

[B91] Tremaroli V. Suzzi C. V. Fedi S. Ceri H. Zannoni D. Turner R. J. (2010). Tolerance of *Pseudomonas pseudoalcaligenes* KF707 to metals, polychlorobiphenyls and chlorobenzoates: effects on chemotaxis-, biofilm- and planktonic-grown cells. *FEMS Microbiol. Ecol.* 74 291–301. 10.1111/j.1574-6941.2010.00965.x 20846140

[B92] Tremaroli V. Workentine M. L. Weljie A. M. Vogel H. J. Ceri H. Viti C. (2009). Metabolomic investigation of the bacterial response to a metal challenge. *Appl. Environ. Microbiol.* 75 719–728. 10.1128/AEM.01771-08 19047385 PMC2632130

[B93] Triscari-Barberi T. Simone D. Calabrese F. M. Attimonelli M. Hahn K. R. Amoako K. K. (2012). Genome sequence of the polychlorinated-biphenyl degrader *Pseudomonas pseudoalcaligenes* KF707. *J. Bacteriol.* 194 4426–4427. 10.1128/JB.00722-12 22843571 PMC3416219

[B94] Tsai Y.-P. Tzeng H. F. Lin J. W. Lu M. S. Lin J-Y. (2013). Verification of enzymes deterioration due to Cu(II) presence in an enhanced biological phosphorus removal system. *Chemosphere* 91 602–607. 10.1016/j.chemosphere.2012.11.080 23347620

[B95] Warnes S. L. Caves V. Keevil C. W. (2012). Mechanism of copper surface toxicity in *Escherichia coli* O157:H7 and *Salmonella* involves immediate membrane depolarization followed by slower rate of DNA destruction which differs from that observed for Gram-positive bacteria. *Environ. Microbiol.* 14 1730–1743. 10.1111/j.1462-2920.2011.02677.x 22176893

[B96] Wiklund S. Johansson E. Sjöström L. Mellerowicz E. J. Edlund U. Shockcor J. P. (2008). Visualization of GC/TOF-MS-based metabolomics data for identification of biochemically interesting compounds using OPLS class models. *Anal. Chem.* 80 115–122. 10.1021/ac0713510 18027910

[B97] Wood T. (1986). Distribution of the pentose phosphate pathway in living organisms. *Cell Biochem. Funct.* 4 235–240. 10.1002/cbf.290040402 3539385

[B98] Wortham B. W. Patel C. N. Oliveira M. A. (2007). Polyamines in bacteria: pleiotropic effects yet specific mechanisms. *Adv. Exp. Med. Biol.* 603 106–115. 10.1007/978-0-387-72124-8_9 17966408

[B99] Ye Y. Wang X. Zhang L. Lu Z. Yan X. (2012). Unraveling the concentration-dependent metabolic response of *Pseudomonas* sp. HF-1 to nicotine stress by ^1^H NMR-based metabolomics. *Ecotoxicology* 21 1314–1324. 10.1007/s10646-012-0885-4 22437205

[B100] Zalasiewicz J. Williams M. Haywood A. Ellis M. (2011). The Anthropocene: a new epoch of geological time? *Philos. Trans. A. Math. Phys. Eng. Sci.* 369 835–841. 10.1098/rsta.2010.0339 21282149

