# Supplementary material for: Metabolomics reveals differences of metal toxicity in cultures of Pseudomonas pseudoalcaligenes KF707 grown on different carbon sources
Source: Front Microbiol. 2015 Aug 17;6:827. doi: 10.3389/fmicb.2015.00827 (PMC4538868; doi:10.3389/fmicb.2015.00827)
Supplement: Supplementary file 1 [file Supplementary_Material.DOCX]

Removal of poor samples and outliers
 GC-MS spectra were initially inspected to identify any samples where excessively low signal was detected. Several samples were removed from this inspection though they all occurred in different sample types leaving at least 4 replicates in all samples. Class based PCA was later used to check for outliers (Supplementary Table 1). Only one model had significant statistics and the outlying sample was removed (Supplementary Figure 3).

Identification of Metabolites
 Data files were converted into netCDF format by MassLynxDatabridge (Waters, Milford, USA). For component selection, AMDIS parameters were set to: Component width: 11, adjacent peak subtraction: Two, Resolution: Low, Sensitivity: High, Shape requirements: High. The GOLM Metabolome Database (GMD)VAR5 library was imported into the NIST MS Search program and components were identified using both the GMD library and the NIST11 Mass Spectral library. Positively identified components were confirmed manually by inspecting the library fragmentation and the fragmentation of representative components extracted from representative chromatograms from each type of sample. This was important as some experimental fragmentation patterns did not match the library fragmentation patterns to a high enough threshold (MS Search probability >90%) to be accepted as positive identifications, despite the retention indices matching exceptionally well (99% similar). In these cases manual inspection allowed fragmentation ions that were causing the decrease in match similarity to be confirmed as true fragmentation products of that compound. For example, malic acid 3TMS has only a small peak for the 117 ion in both the NIST and GMD library, however this ion was the dominant ion in experimental samples. Since this ion corresponds to a carboxylic acid group with a TMS group replacing the removed proton, of which malic acid can produce two such ions it was concluded that this component was indeed malic acid 3TMS. Hexose-6-phosphate was the name given to an ambiguous metabolite that may have been glucose-6-phosphate or mannose-6-phosphate as this difference cannot be resolved by GC-MS. While confirming all positive identifications a custom library of fragmentation patterns was generated that included all identified compounds from all sample types as well as reproducible but unknown components. At completion this library contained 489 spectra including both identified and unidentified true and artifactual compounds.

Removal of Derivatization Artifacts

Artifacts were removed as described in the main text. For a complete list of artifacts, their retention times and intensities, see supplementary Table 1.

Quantification of Metabolites

MET-IDEA parameters: Chromatography: GC, Average peak width: 0.05, Minimum peak width: 1, Maximum peak width: 3, Peak start/stop slope, Adjusted retention time accuracy: 1, Peak overload factor: 0.9. These parameters were determined manually to ensure that adjacent peaks would not contribute to peak quantification.

Confirmation of Analyte Quantification Quality

Toensurethattheionsthathadbeenquantifiedwererepresentativeofthatanalyse, the sum of all ions that had been pre-selected for each analyte was determined. The correlation between the ions that were part of this sum and the sumwasthendeterminedacrossall samples. If the mean correlation was below 0.8 then these ions were determined to not be representative enough of that analyte and so quantification of the analyte was deemed inaccurate and was excluded from further analysis. This ensured that the sum of quantified ions that was used to represent each analyse was robust and only analytes that were present in enough samples to generate a strong correlation were used.

Data Analysis: Pre-processing
 Probabilistic quotient normalization (PQN) procedure: the data were divided into four blocks, one for each type of sample. These blocks were biphenyl cells, succinate cells, biphenyl spent media and succinate spent media. Block based normalization allows for the data to be normalized more accurately as the correction factor is specific for each of the disparate sample types. Within each block an ‘average’ spectrum was determined by taking the median of each analyte. For each sample the difference ratio between the ‘average’ spectrum was determined for each analyte. The median of these difference ratios was then determined and used as correction factor for all analytes in that sample. The median was used instead of the mean as it is less sensitive to outlying high or low values. After normalization the blocks were recombined, data were log transformed and each analyte was mean-centered and scaled to unit-variance. These transformations allow variables with disparate dynamic ranges and means to be compared on the same scale.

Identification of Unknown Analytes
 Biological interpretation of the data implied the possibility of several metabolites that had not been identified, and could not be identified due to their lack of standards available in either Golm or NIST libraries. Predicted compounds were drawn in Mass Spec Calculator Pro V 4.09 (ChemSW,Inc/Quadtech Associates, Ringoes, USA) and hydroxyl and amine groups had their free hydrogens replaced with trimethylsilyl (TMS) groups. The previously generated library of unknown analytes was then searched for analytes which had their largest m/z at calculated m/z of the unfragmented derivitized possible analyte. Additionally an m/z at M-15 of the mass ion was used as a pre-screening ion to identify unknowns that could possibly be the derivitized compound of interest as the loss of a single methyl group is a common fragmentation. Unknown analytes which had m/z peaks that matched the M and M-15 masses of a compound being searched for were further checked for m/z fragmentation peaks that matched possible fragmentation outcomes of the known, derivitized compound drawn in Mass Spec Pro. By this process only 3 unknown analytes were given probable identifications. The analytes that these unknowns were hypothesized to be were 2-hydroxymuconic semialdehyde (2TMS) and 2-phosphoglycolic acid (3TMS). After confirming the hypothetical fragmentation matched the observed m/z peaks of these unknown analytes, the Kovát’s retention indexof these compounds were calculated and compared to the unknown analyte’s. By this process Unknown RT:11.37 was identified as 2-hydroxymuconic semialdehyde 2TMS. Unknown RT:11.41 which was immediately adjacent to this peak, with an almost identical fragmentation pattern is likely the same analyte. Unknown RT:11.58 was identified as unequivocally as phosphoglycolic acid 3TMS. See the supplementary identification of unknowns for the mass spec fragmentation patterns.

Use of p(corr) to determine correlation between metabolites and sample type
 p(corr) indicates the degree of correlation between a metabolite and a component. In all pairwise models that were generated in this study, there was only a single predicted component, upon which samples of different types were consistently separated. This means that the correlation with the predicted component corresponds to a correlation with a sample type. The particular sample type for each model was determined using the corresponding scores plots for that model as it is arbitrary whether the control class is assigned to the positive or negative side of a component when the OPLS-DA model is generated (Supplementary Figures 4 and 5). Thus p(corr) was used as an indication of whether a metabolite was correlated with control or metal exposed samples.

Generation and Interpretation of Secreted Metabolites SUS plots

Metabolites were quantified from the spent media of cultures, but the same metabolites that were quantified from within cells were also found in the spent media. To determine which metabolites were actually altered in the spent media not just due to changes in the cells spilling over to the spent media, VIP and p(corr) values from both models comparing control and exposed cells and spent media were considered together. Metabolites were considered to be secreted only if their VIP was above 0.8 in the spent media model (indicating a significant difference between the control and exposed samples). From this reduced list of metabolites only those with either a VIP below 0.8 in the cells (indicating no significant difference between the control and exposed cells meaning the difference in the spent media must be due to changes in secretion) or if the p(corr) in the spent media model was opposite that of the cells model. Metabolites that increased in the spent media but decreased in the cells in response to metal exposure must have been secreted more in response to the metal whereas those that decreased in the spent media but increased in the cells in response to metal exposure must have been secreted less in response to metal exposure. By indirectly comparing the data in this fashion it precludes the need to directly compare the cells to the spent media samples which cannot be done accurately as only a portion of the spent media was analyzed while all of the cells from a sample were collected and analyzed.

Pathway Enrichment Analysis
 *Pseudomonas aeruginosa* PA7 was used to generate the background set of metabolites as according to phylogenetic analysis (Triscari-Barberi, unpublished results) and BLAST searches of tRNA synthetases this is *P. pseudoalcaligenes*KF707’s closest relative that was available in the database. Metabolic pathways that are commonly found by this type of analysis due to the inclusion of large numbers of metabolites in their pathway (metabolic pathways, ABC transporters, phospho-transfer system, two component system, cyanoaminoacid metabolism) as well as pathways that obviously erroneously enriched (carbazole degradation, toluene and xylene degradation, methane metabolism and biosynthesis of siderophore group non-ribosomal peptides) were excluded. Pathways were then further screened for relevance to ensure that the affected metabolites that were implicating a pathway would actually be involved in that pathway in *Pseudomonas pseudoalcaligenes* KF707. By this screening, glutathione metabolism (implicated metabolites are only involved in trypanosomatid glutathione metabolism), oxidative phosphorylation (this pathway only includes 9 metabolites making its enrichment guaranteed by only succinate and fumarate being affected), lysine degradation (this pathway would only be expected in a culture growing on lysine or peptides containing lysine, not on a defined media with ammonia as the sole nitrogen source and carbon coming from only a single source, additionally, the metabolites that implicated this pathway were mostly disjointed from one another), phenylalanine metabolism (implicated by benzoic acid and salicylic acid, which can be derived from phenylalanine but in the biphenyl grown cultures that this pathway was implicated in these compounds obviously were derived from biphenyl degradation not phenylalanine), and butanoate metabolism (implicated mainly by Krebs’ cycle intermediates present in this pathway) pyrimidine metabolism (only Cu, both β-alanine and propanedioic acid implicated but these are only uracil degradation products in other organisms).

Supplementary Table 1: Analytes present in derivitization control samples. Retention time (RT), retention index (RI) and peak area are mean of all derivitization control samples run.

| Name | RT | RI | Area |
| --- | --- | --- | --- |
| Low MW RT:5.0 | 5.004 | 981.7 | 266375 |
| Unknown Low MW RT: 5.1 | 5.1137 | 991.4 | 3573868 |
| Low MW RT:5.14 | 5.1456 | 994.3 | 332613.5 |
| Low MW RT:5.17 | 5.1692 | 996.4 | 142543 |
| Tetrasiloxane | 5.3549 | 1012.9 | 14647 |
| Unknown: Bp_2 RT:5.51 | 5.4008 | 1016.9 | 16864 |
| 2-Hydroxy pyridine | 5.4331 | 1019.8 | 22978 |
| Unknown RT:5.58 | 5.5786 | 1032.7 | 35919 |
| N,N-Diethylcarbamate 1TMS | 5.6755 | 1041.3 | 104711.5 |
| Unknown: Bp_3 RT:5.74 | 5.7397 | 1047 | 11248 |
| Unknown RT:5.96 | 5.9577 | 1066.4 | 352625.5 |
| Unknown RT: 6.0 | 6.0097 | 1071 | 272351.5 |
| Unknown RT:6.17 | 6.1778 | 1085.9 | 146841 |
| Unknown RT:6.2 | 6.2264 | 1090.3 | 286432 |
| Unknown RT:6.24 | 6.2424 | 1091.7 | 170877.5 |
| Unknown RT:6.31 | 6.308 | 1097.5 | 58428 |
| Unknown RT:6.5 | 6.487 | 1113.4 | 291267 |
| Unknown Silane RT:6.8 | 6.6897 | 1131.4 | 1331628 |
| Unknown RT:6.78 | 6.7797 | 1139.4 | 66098.5 |
| Pentasiloxane | 6.9637 | 1155.7 | 14305 |
| Unknown: Bp_M_3_R RT:7.60 | 7.0553 | 1163.9 | 4447 |
| Unknown RT:7.17 | 7.1658 | 1173.7 | 3210 |
| Dodecane | 7.474 | 1201.1 | 8887 |
| Unknown: Bp_2 RT:5.33 | 7.5199 | 1205.1 | 980 |
| 2-siloxyltetrasiloxane | 7.65 | 1216.7 | 2174 |
| Silanamine 3TMS | 7.7668 | 1227.1 | 39694 |
| Unknown RT:7.841 | 7.8412 | 1233.7 | 31394 |
| Unknown RT:7.94 | 7.9423 | 1242.7 | 112864.5 |
| Pentadecane | 8.347 | 1278.6 | 73379.5 |
| Unknown RT:8.349 | 8.347 | 1278.6 | 55980.5 |
| Unknown RT:8.450 | 8.4495 | 1287.7 | 8010 |
| Unknown RT:8.65 | 8.6511 | 1305.6 | 480136.5 |
| Unknown RT:8.85 | 8.8458 | 1322.9 | 369826 |
| Unknown RT:8.89 | 8.8827 | 1326.2 | 122777.5 |
| Unknown: (Similar to Lumichrome) | 8.9492 | 1332.1 | 16036 |
| Uracil 2TMS | 9.0173 | 1338.1 | 7111 |
| Unknown RT:9.09 | 9.2064 | 1354.9 | 25942 |
| Unknown RT:9.77 | 9.7706 | 1405 | 1513 |
| Unknown RT:9.96 | 9.9518 | 1421.1 | 358892 |
| Unknown RT:10.26 | 10.2603 | 1448.5 | 19447 |
| Unknown RT:10.37 | 10.3658 | 1457.9 | 44617 |
| Unknown RT:10.43 | 10.4359 | 1464.1 | 1743 |
| Unknown RT:10.46 | 10.4559 | 1465.9 | 64633 |
| Unknown RT:10.56 | 10.5566 | 1474.8 | 107840 |
| Unknown RT:13.40 | 10.5749 | 1476.5 | 18212 |
| Unknown: ScP_M_1_No_D RT:10.60 | 10.5965 | 1478.4 | 17589 |
| Unknown RT:10.71 | 10.7055 | 1488.1 | 27184 |
| Silanamine | 10.841 | 1500.1 | 14155 |
| Unknown RT:11.10 | 11.0977 | 1527.6 | 83237 |
| Similar to Tertbutylphenol RT:11.2 | 11.1959 | 1538.1 | 523882.5 |
| Unknown RT:11.28 | 11.2782 | 1546.9 | 98424 |
| Unknown RT:11.38 | 11.3793 | 1557.7 | 111028 |
| Unknown RT:11.49 | 11.4924 | 1569.8 | 11403 |
| Unknown RT:11.59 | 11.592 | 1580.4 | 27401 |
| Dodecanoic acid TMS | 12.3225 | 1658.5 | 15939 |
| Unknown RT:12.42 | 12.4181 | 1668.7 | 75076 |
| Unknown RT:12.79 | 12.7909 | 1708.6 | 26676 |
| Unknown RT:13.06 | 13.062 | 1737.6 | 64977 |
| Unknown RT:13.15 | 13.145 | 1746.5 | 40132 |
| Unknown RT:13.20 | 13.1951 | 1751.8 | 47849 |
| Unknown RT:13.283 | 13.2825 | 1761.2 | 38927 |
| Unknown RT:13.32 | 13.3231 | 1765.5 | 122705 |
| Unknown RT:13.51 | 13.3976 | 1773.5 | 142681 |
| Unknown RT:13.46 | 13.4547 | 1779.6 | 11698 |
| Unknown RT:15.01 | 13.5668 | 1791.6 | 6147 |
| Unknown RT:13.61 | 13.6118 | 1796.4 | 68969 |
| Unknown RT:13.76 | 13.7553 | 1811.7 | 2588 |
| Unknown RT:14.76 | 14.7564 | 1922.2 | 229053 |
| Unknown RT:14.94 | 14.945 | 1946 | 5975 |
| Hexadecanoic acid TMS | 15.7499 | 2047.7 | 3543529 |
| Heptadecanoic acid TMS | 16.5057 | 2143.1 | 100858 |
| Octadecanoic acid TMS | 17.2506 | 2243 | 8267357 |
| Unknown RT:18.63 | 18.6279 | 2444.1 | 66502.5 |
| Unknown RT:18.99 | 18.986 | 2496.3 | 171614 |
| Unknown High MW RT:21.3 | 21.2651 | 2865.4 | 441333 |
| Unknown RT:21.27 | 21.2656 | 2865.5 | 259353 |
| Unknown RT:22.89 | 22.8897 | 3140.2 | 2069168 |
| Unknown RT:28.07 | 28.0598 | 4014.6 | 132886.5 |


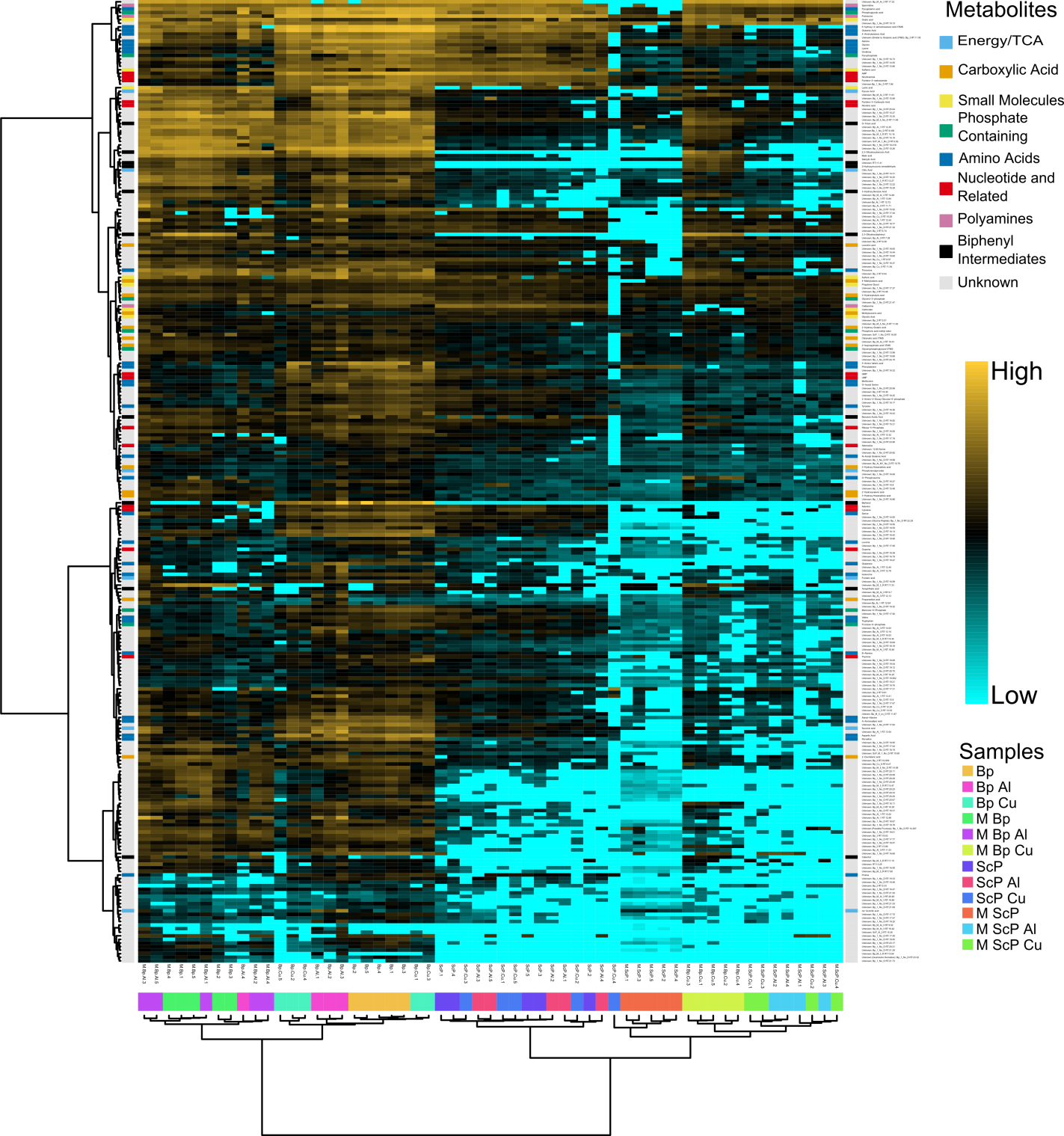

Supplementary Figure 1: Hierarchical clustering analysis and heatmap of GC-MS quantified metabolites from cells and spent media of cultures of *Pseudomonas pseudoalcaligenes* KF707 grown on succinate or biphenyl as the sole carbon source under control, Aluminum or Copper exposure. Data were only log transformed. Gold indicates high concentrations of metabolite while cyan indicates low, black being average (across ALL samples) for that metabolite. Distances between samples and variables were determined using euclidean distance and clusters (both of metabolites and samples) were determined by Ward’s method. The color bars at the end of the sample dendrogram indicate the sample type. Color bars at the end of the metabolite dendrogram and beside the metabolite names indicate general metabolic class.


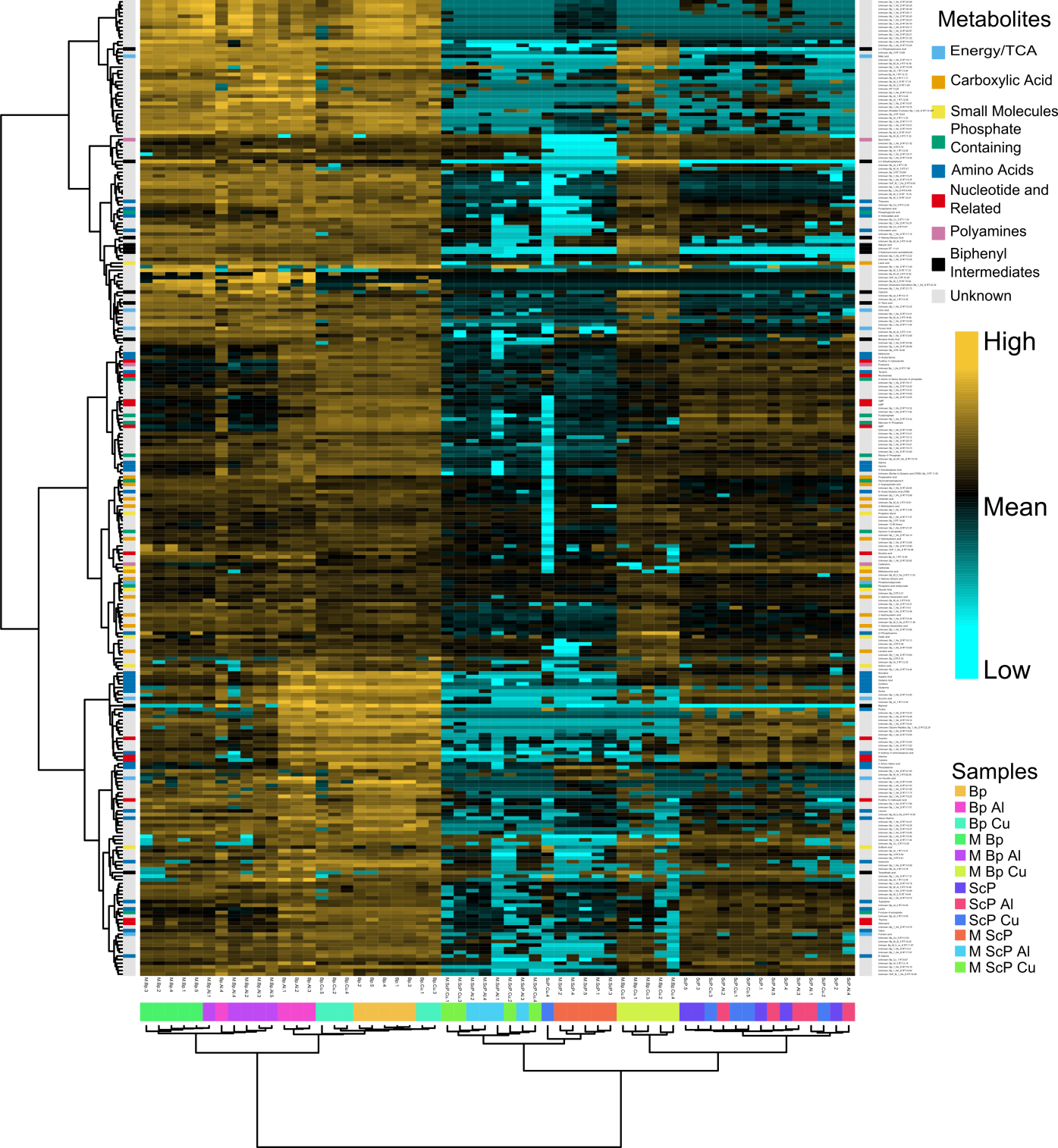


Supplementary Figure 2: Hierarchical clustering analysis and heatmap of GC-MS quantified metabolites from cells and spent media of cultures of *Pseudomonas pseudoalcaligenes* KF707 grown on succinate or biphenyl as the sole carbon source under control, Aluminum or Copper exposure. Data were normalized, mean-centered and scaled to unit variance to allow comparison of metabolites with varying dynamic ranges and variances. Gold indicates high concentrations of metabolite while cyan indicates low, black being average (across ALL samples) for that metabolite. Distances between samples and variables were determined using euclidean distance and clusters (both of metabolites and samples) were determined by Ward’s method. The color bars at the end of the sample dendrogram indicate the sample type. Color bars at the end of the metabolite dendrogram and beside the metabolite names indicate general metabolic class.

Supplementary Table 2: Model statistics of PCA-Class models showing intraclass variation of GC-MS metabolomics quantification of samples from cells and spent media of cultures of *Pseudomonas pseudoalcaligenes* KF707 grown on either biphenyl or succinate as the sole carbon source and exposed to either control, Aluminum or copper.

| Model | A | N | R^2^ | Q^2^ |
| --- | --- | --- | --- | --- |
| PCA-Class(Bp) | 1 | 5 | 0.335 | -0.1 |
| PCA-Class(Bp Al) | 1 | 4 | 0.586 | 0.0114 |
| PCA-Class(Bp Cu) | 1 | 5 | 0.464 | -0.0057 |
| PCA-Class(M Bp) | 1 | 5 | 0.377 | -0.0924 |
| PCA-Class(M Bp Al) | 2 | 5 | 0.686 | -0.21 |
| PCA-Class(M Bp Cu) | 1 | 5 | 0.447 | -0.0642 |
| PCA-Class(M Sc) | 1 | 5 | 0.408 | 0.0078 |
| PCA-Class(M Sc Al) | 1 | 4 | 0.545 | -0.1 |
| PCA-Class(M Sc Cu) | 1 | 4 | 0.425 | -0.1 |
| PCA-Class(Sc) | 1 | 5 | 0.397 | -0.1 |
| PCA-Class(Sc Al) | 1 | 5 | 0.417 | -0.1 |
| **PCA-Class(Sc Cu)** | **1** | **5** | **0.723** | **0.581** |


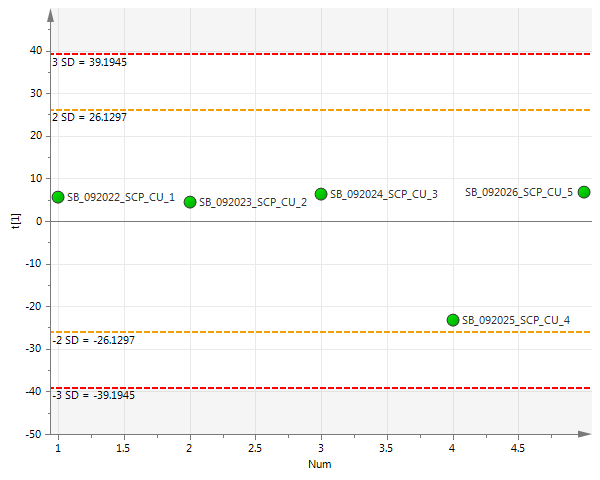

Supplementary Figure 3: PCA Scores plot from PCA-Class model of GC-MS metabolomic quantification of samples from cells of *Pseudomonas pseudoalcaligenes* KF707 grown on succinate as the sole carbon source and exposed copper.


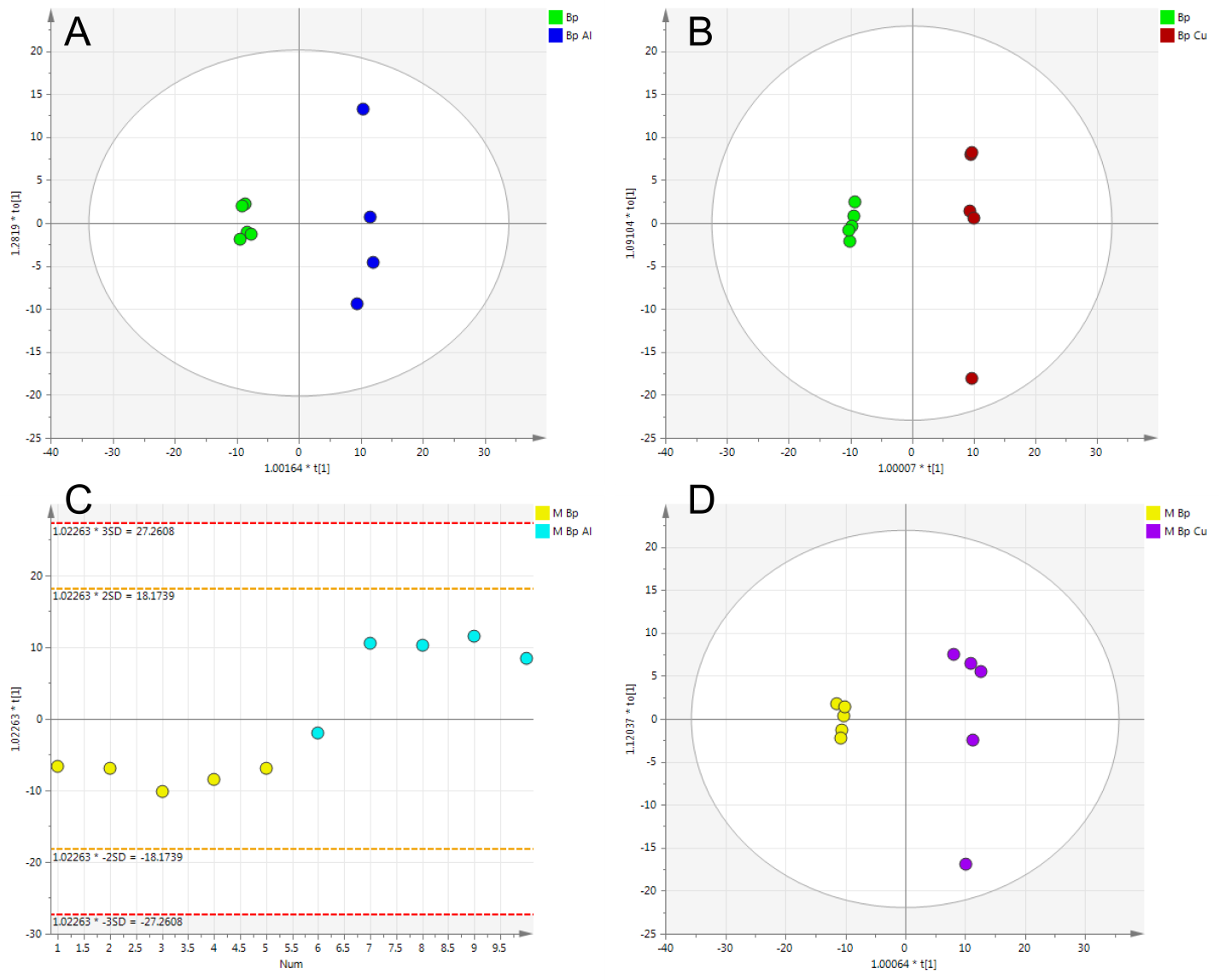
Supplementary Figure 4: OPLS-DA Scores Plots from pairwise models comparing GC-MS metabolomic quantification of samples from cells and spent media of cultures of *Pseudomonas pseudoalcaligenes* KF707 grown on biphenyl as the sole carbon source and exposed to either control, aluminum or copper.


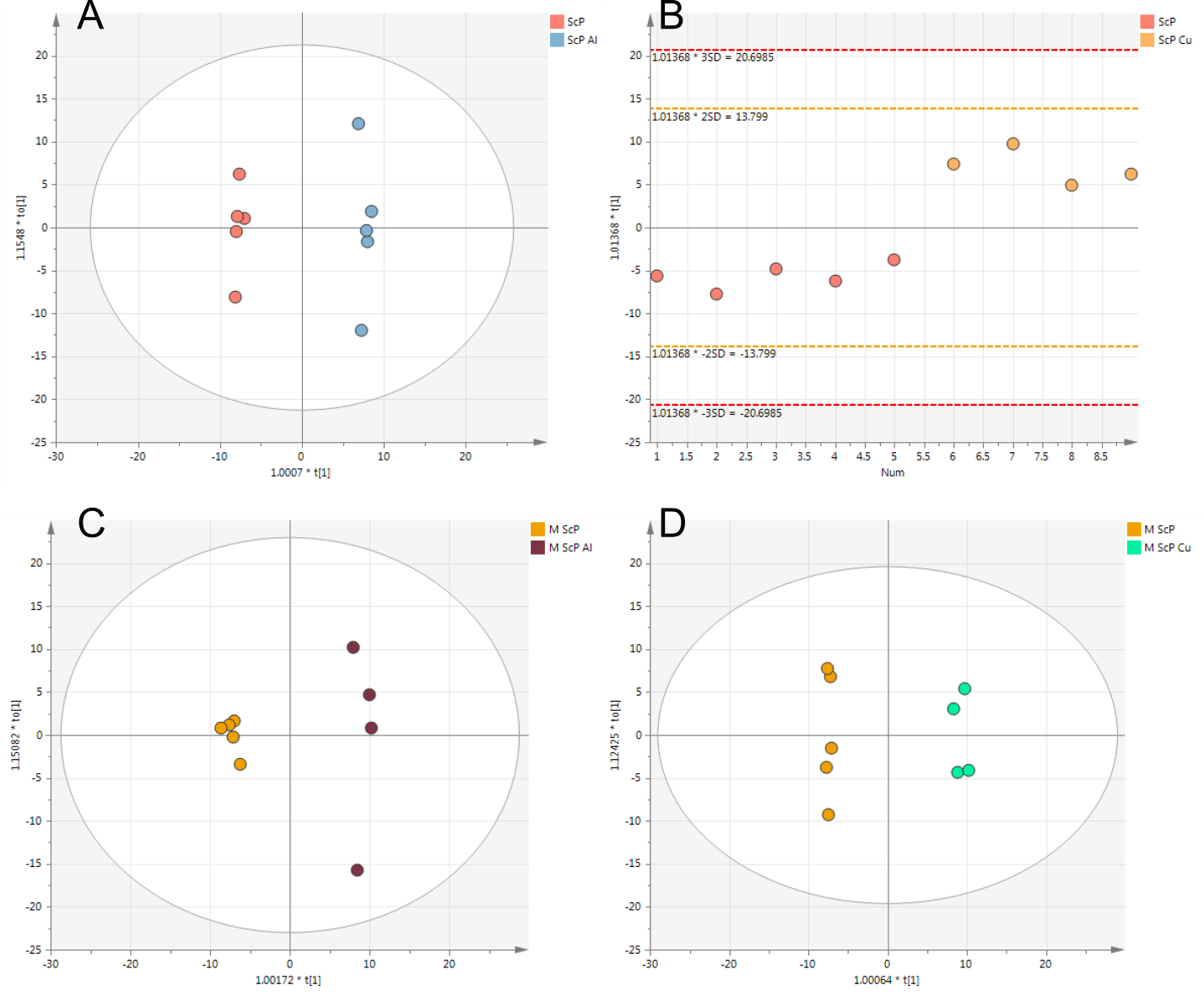

Supplementary Figure 5: OPLS-DA Scores Plots from pairwise models comparing GC-MS metabolomic quantification of samples from cells and spent media of cultures of *Pseudomonas pseudoalcaligenes* KF707 grown on succinate as the sole carbon source and exposed to either control, Aluminum or copper.


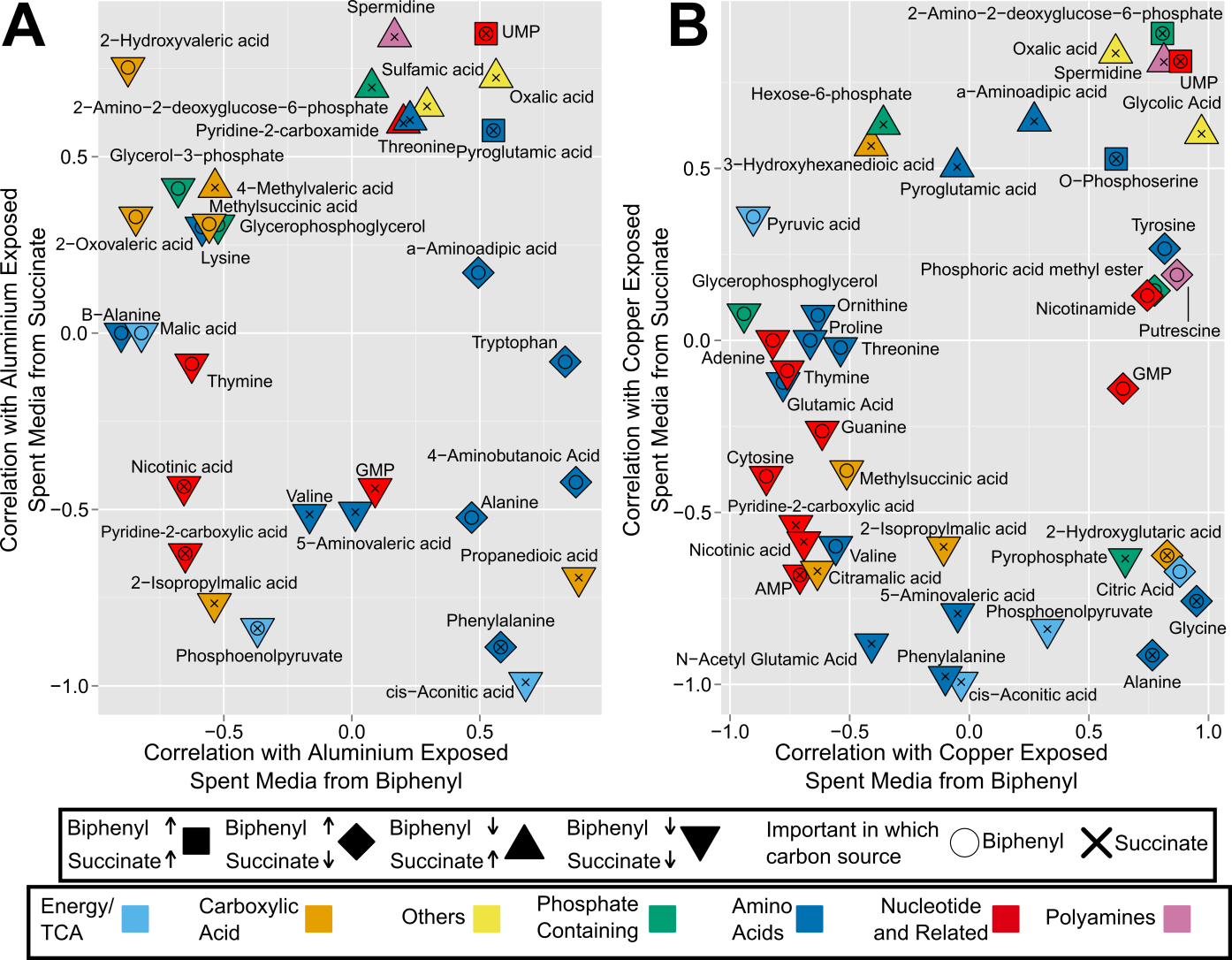

Supplementary Figure 6: Shared and unique structures plots comparing extracellular metabolic changes of identified metabolites caused by Aluminum (A) and Copper (B) in cultures of *Psedomonas pseudoalcaligenes* KF707 grown on either succinate or biphenyl as the sole carbon source. The data plotted here were derived from pairwise OPLS-DA models that compared control to metal exposed samples for each carbon source. Coordinates were determined by the p(corr) of each metabolite from each model, only those with a VIP>0.8 in at least one media type were plotted. Metabolites with a VIP>0.8 in biphenyl models are marked with a circle, in succinate an X. The larger the p(corr) the greater the correlation with metal exposure for that media type. To identify metabolites that were being secreted, data from the OPLS-DA models comparing the control and metal exposed cells samples for each media type were also used. Metabolites were determined to be secreted if the VIP was above 0.8 in the spent media models and either the VIP was below 0.8 in the cells models or the p(corr) was opposite what was observed in the spent media. I.E. if a metabolite was found to change significantly in both the cells and spent media and was correlated with the metal exposed samples in the media and the control in the cells, this metabolite was secreted in response to metal exposure. Conversely, if the opposite was true, i.e. the metabolite was correlated with the control samples in the spent media and the metal exposed cells, this metabolite’s secretion decreased in response to metal exposure. The association of each metabolite with control or metal exposed cell samples was determined using p(corr), which indicates the degree of correlation of the metabolite with a sample type. Actual values were not used, only the directionality (i.e. above or below zero) as metabolites with high (>0.8) VIP scores tended to have high p(corr) absolute values. Using these values metabolites were assigned a shape that indicates how secretion of the metabolite was altered by metal exposure: squares were secreted more in both biphenyl and succinate, diamonds were secreted more in biphenyl but less in succinate, upwards pointed triangles were secreted more in succinate but less in biphenyl and downwards pointed triangles were secreted less in both in response to metal exposure. Metabolites were broadly classified according to chemical structure and/or biological function: those from the TCA cycle and energy producing pathways (light blue), other carboxylic acids (orange), small molecules (yellow), phosphate containing (green) (A and C), amino acids (dark blue), nucleotides and related pyridine/pyrimidine compounds (red) and polyamines (purple) (B and D). The top two panels show the effects of Aluminum (A and B) and the bottom two copper (C and D).


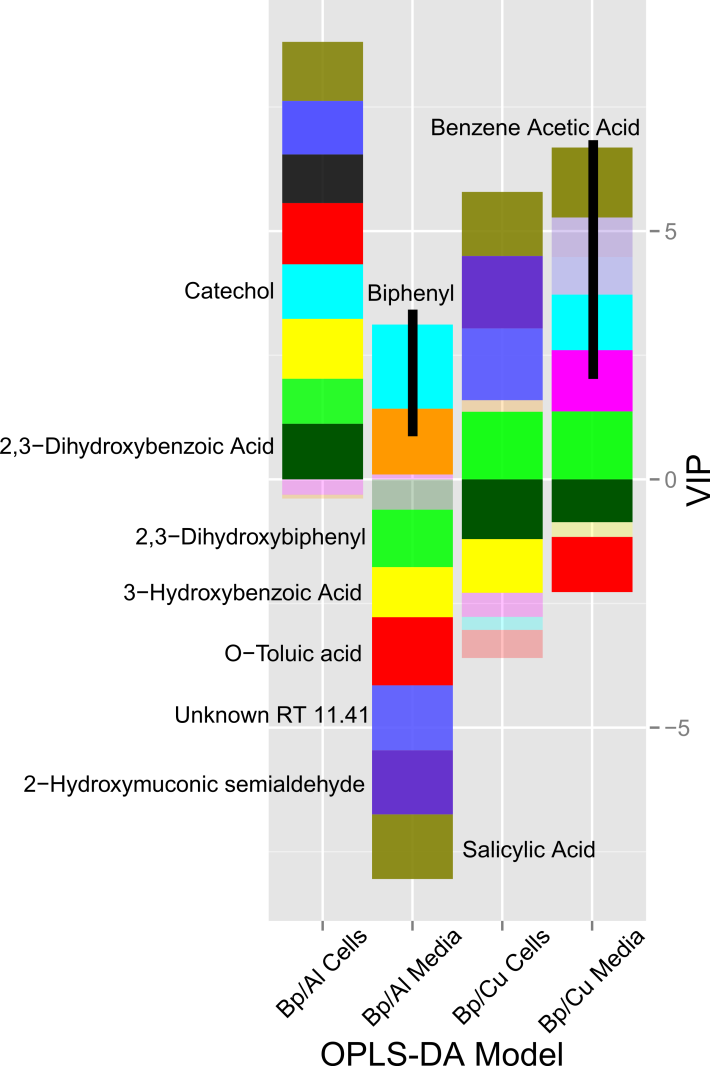


Supplementary Figure 7: Variable Influence on Projection (VIP) of metabolites related to degradation of biphenyl from OPLS-DA models comparing control to metal exposed cells and spent media of *Pseudomonas pseudoalcaligenes* KF707 grown on biphenyl as a sole carbon source. Positive VIP values indicate a positive p(corr) value meaning an increase in metal exposed samples, negative a decrease. Transparent bars indicate a VIP<0.8, not significantly changed. Metabolites categorized as relating to biphenyl degradation are: biphenyl (orange), 2,3-dihydroxybiphenyl (light green), 2,3-dihydroxybenzoic acid (dark green), 3-hydroxybenzoic acid (yellow), salicylic acid (brown), benzene acetic acid (pink), o-toluic acid (red), catechol (cyan), 2-hydroxymuconic semialdehyde (purple), unknown RT:11.41 (light purple).

Identification of Unknown RT 11.58 as 2-Phosphoglycolic acid (3TMS)

Table 1: Predicted fragmentation of 2-Phosphoglycolic acid (3TMS)

| Ion | Mass | Loss | Loss Identity |
| --- | --- | --- | --- |
| Mass | 372 | 0 | - |
| Methyl loss | 357 | 15 | Methyl |
| O-silyl and =O loss | 267 | 105 | O-silyl and =O |
| O-Methyl (-H) phosphate 2TMS | 255 | 117 | Carboxylic TMS |
| O-Methyl (-H) phosphate 2TMS -=O | 239 | 133 | Carboxylic TMS and =O |
| Phosphate 2TMS –O | 225 | 147 | Glycolic acid (-H) TMS |
| O-Methyl (+H) phosphate (-O) 1TMS | 167 | 205 | Carboxylic TMS and O-silyl |
| O-Methyl phosphate (-O) 1TMS –methyl | 151 | 221 | Carboxylic TMS and O-silyl and methyl |
| 2TMS OR Glycolic acid(-H) TMS | 147 | 225 | Mass -2TMS OR Phosphate 2TMS –O |
| 1TMS | 73 | 299 | Mass –TMS |


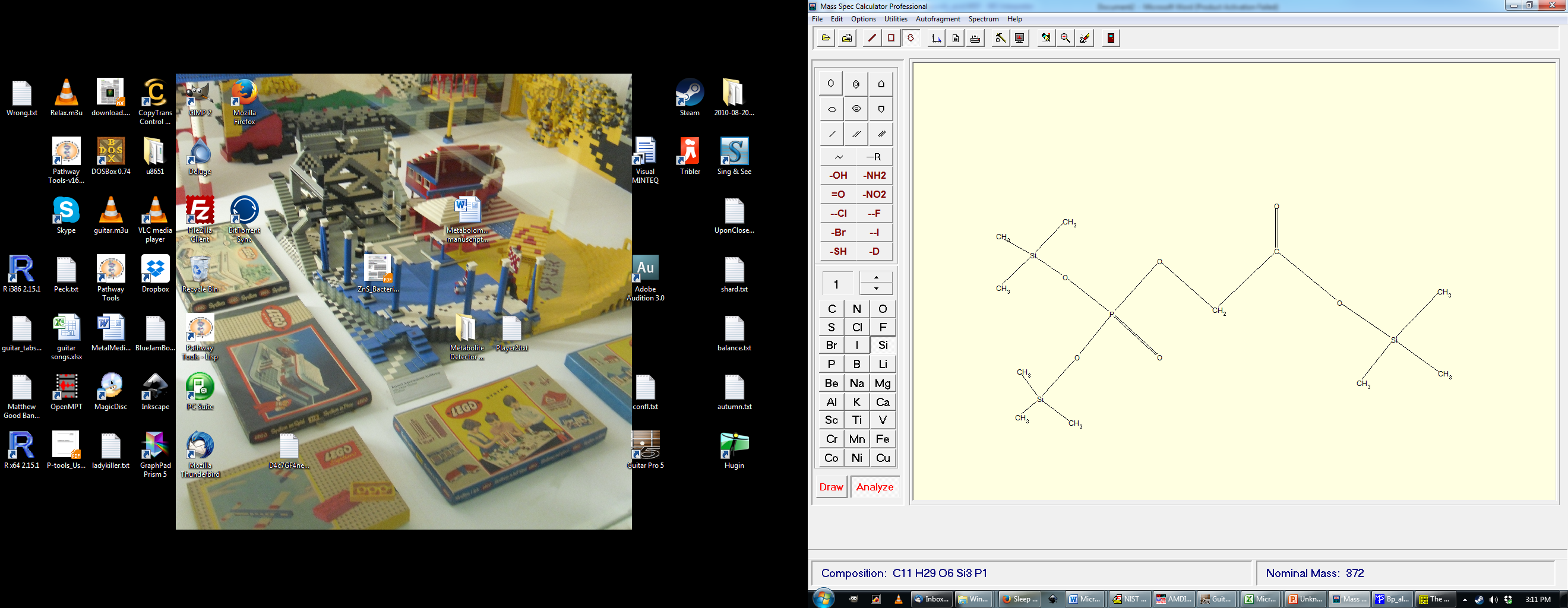
Figure 1: Structure of 2-phosphoglycolic acid 3TMS.


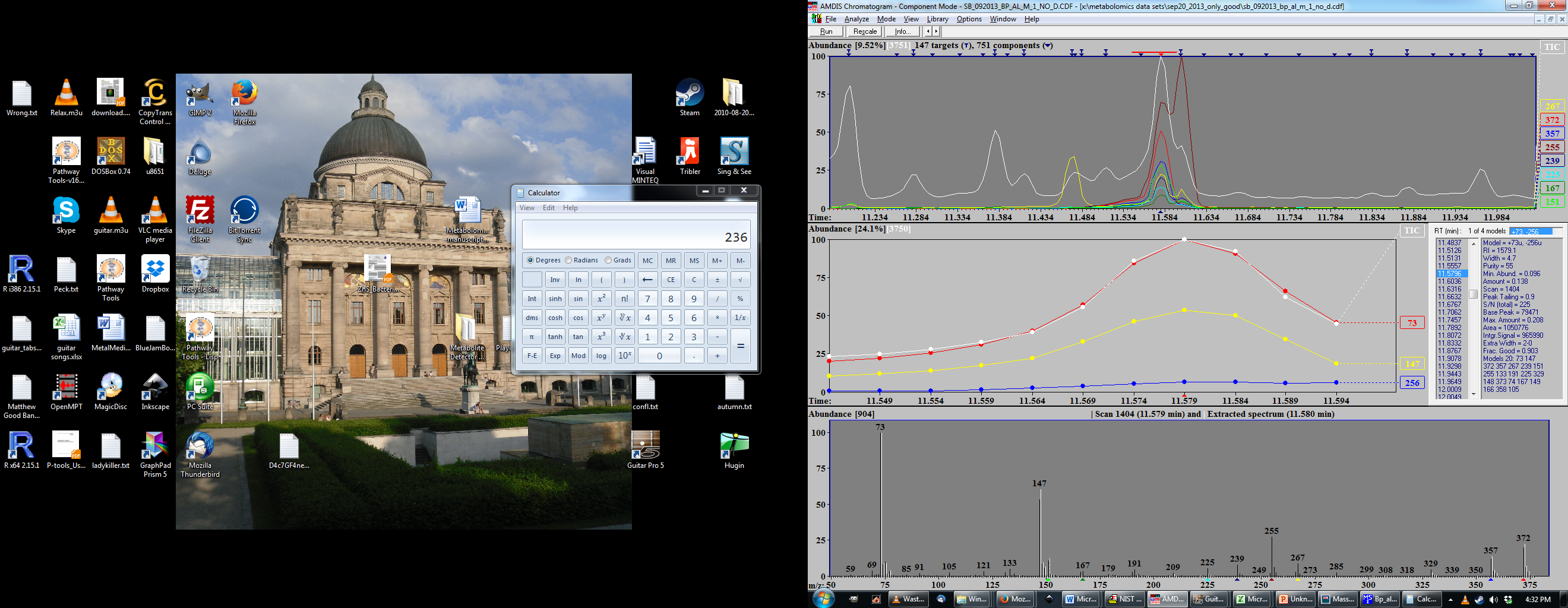
Figure 2: Gas chromatogram showing peak at RT:11.5787 (RI:1579). Peak is composed of ions 267 (yellow), 372 (red), 357 (blue), 255 (dark red), 239 (dark blue), 225 (cyan), 167 (dark green), 151 (light green).


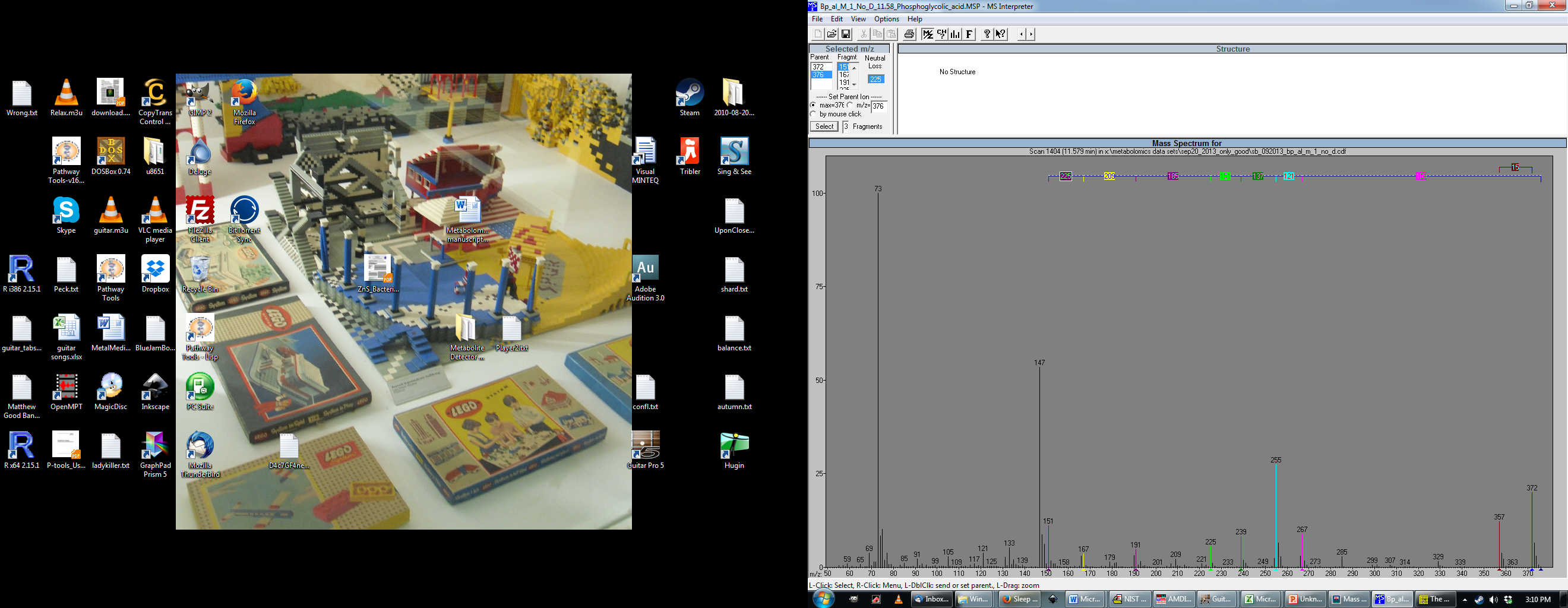
Figure 3: Mass spectrum of unknown RT 11.58.

Table 2: Functional group analysis to determine retention index of 2-phosphoglycolic acid (3TMS). Carboxyl group was interpreted as one >CO group and one –O– group.

| Group | Number of instances | Value | Sum |
| --- | --- | --- | --- |
| -CH3 | 9 | 112 | 1008 |
| >CH2 | 1 | 99 | 99 |
| >CO | 1 | 253 | 253 |
| >Si< | 3 | -115 | -345 |
| >PO- | 1 | 246 | 246 |
| -O- | 4 | 75 | 300 |
| Total | x | x | 1561 |

Table 3: Functional group analysis to determine retention index of 2-phosphoglycolic acid (3TMS). Carboxyl group was interpreted as one –CO-O-.

| Group | Number of instances | Value | Sum |
| --- | --- | --- | --- |
| -CH3 | 9 | 112 | 1008 |
| >CH2 | 1 | 99 | 99 |
| -CO-O- | 1 | 266 | 266 |
| >Si< | 3 | -115 | -345 |
| >PO- | 1 | 246 | 246 |
| -O- | 3 | 75 | 225 |
| Total | x | x | 1499 |

2-Phosphoglycolic acid was drawn and derivitized to 2-phosphoglycolic acid (3TMS) (Figure 1) and fragmentation predicted (Table 1). Deconvoluted, calibrated GC spectra were searched for peaks that had all 8 predicted fragementation ions. Unknown RT:11.58 was identified as containing all predicted fragments (Figures 2 and3). The Kovát’s retention index (Stein et al., 2007) was predicted for 2-phosphoglycolic acid (3TMS) using two possible interpretations of the functional groups present (Tables 2 and3). The mean of these twoestimates was RI: 1530. This is very similar to the retention index of Unknown RT:11.58 of 1579, only differing by 49 units. As a comparison 2-hydroxyglutaric acid (3TMS) has an estimated RI of 1489 but the NIST library value is 1572, a difference of 83 units. Between this very good match and the excellent coherence between the predicted and actual fragmentation patterns unknown RT:11.58 was concluded to be positively identified as 2-phosphoglyocolic acid (3TMS).

Identifcation of Unknown RT:11.37 as 2-Hydroxymuconic semialdehyde (2TMS)

Table 4: Predicted fragmentation of 2-hydroxymuconic semialdehyde(2TMS)

| Ion | Mass | Loss | Loss Identity |
| --- | --- | --- | --- |
| Mass | 286 |  |  |
| Mass –methyl | 271 | 15 | Methyl |
| Mass –methyl – CH=O (+H) | 243 | 43 | Methyl and CH=O (+H) |
| Mass –methyl –methyl –CH=O | 227 | 59 | 2Methyl and CH=O |
| Mass –TMS –H | 196 | 90 | TMS+H |
| Mass –carboxylic TMS | 169 | 117 | Carboxylic TMS |
| Mass –carboxylic TMS -=O | 153 | 133 | Carboxylic TMS and =O |
| C-C=CH-O-TMS(-methyl) | 111 | 175 | O-TMS and =O and O=CH-CH=CH and methyl |
| CH=C-OC-O=O | 85 | 201 | 2TMS O=CH-CH=CH |
| CH-CH=CH-CH=C-C (-H) | 75 | 211 | 2 O-TMS and 2=O |
| 2TMS | 147 | 139 | Mass -2TMS |
| 1TMS | 73 | 213 | Mass-1TMS |


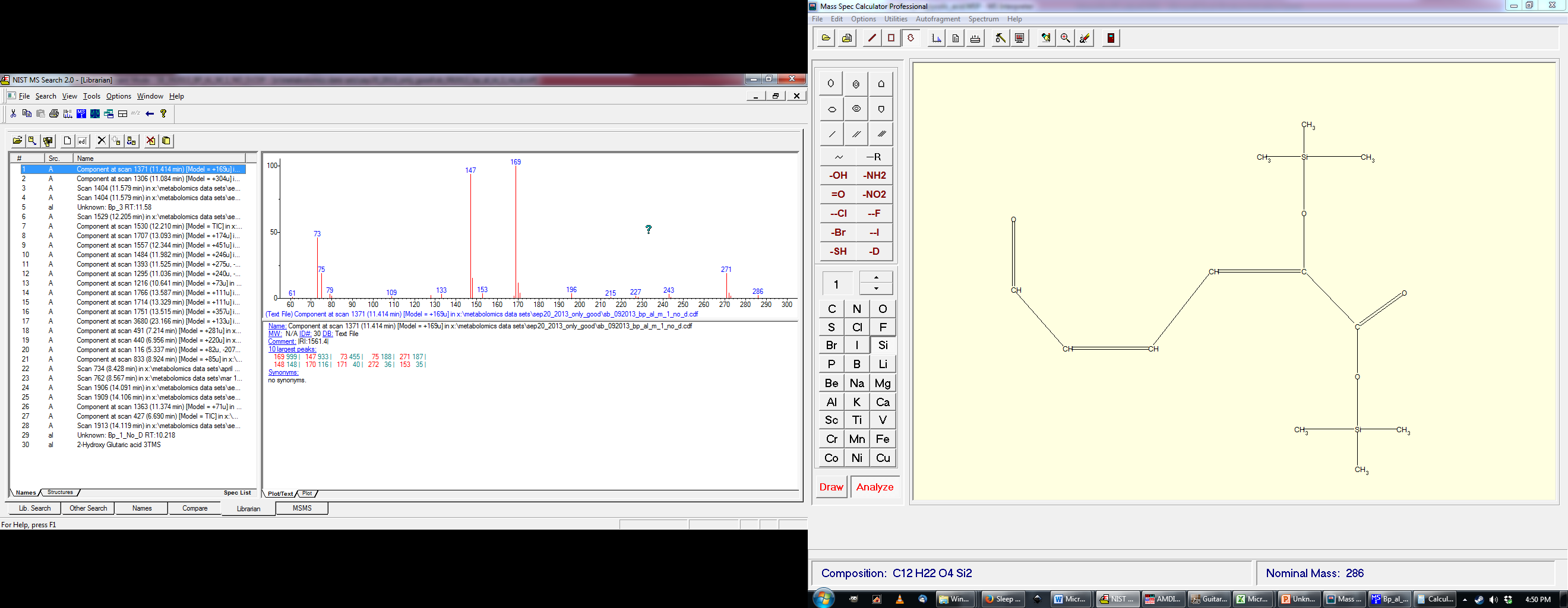

Figure 4: Structure of 2-hydroxymuconic semialdehyde.

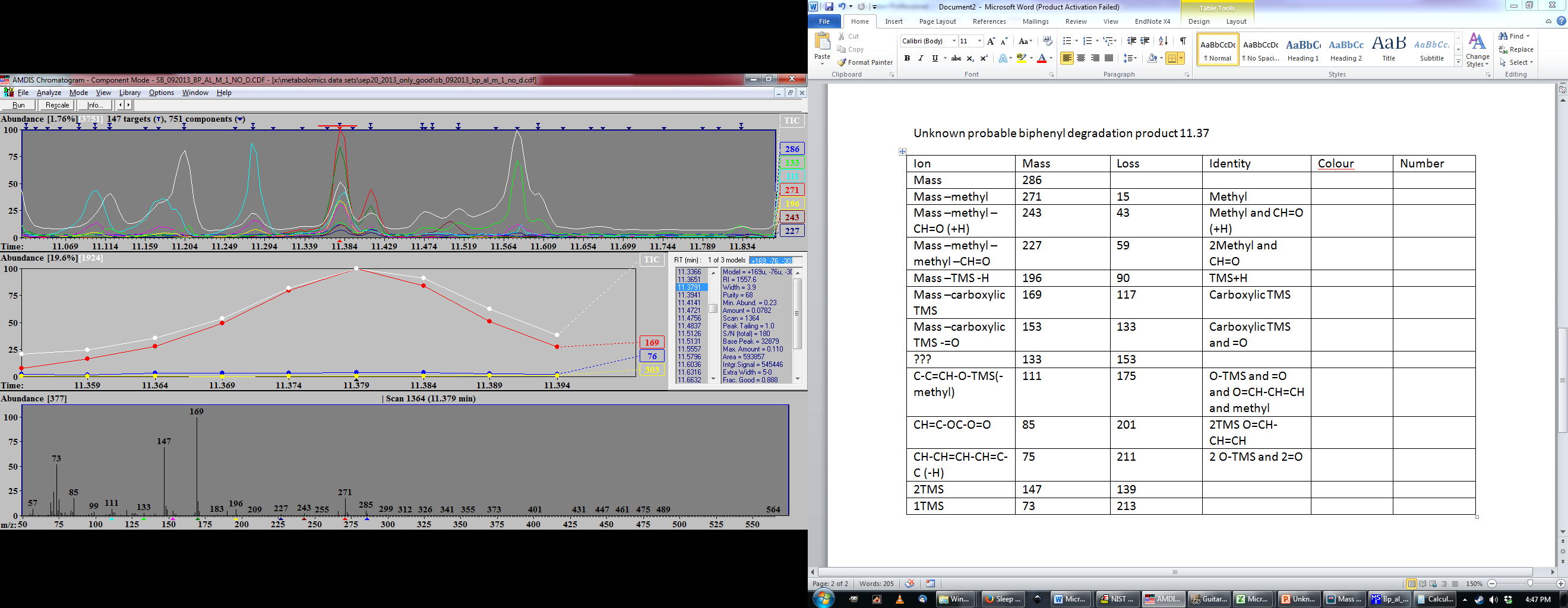
Figure 5:Gas chromatogram showing peak at RT:11.37 (RI:1557). Peak is composed of ions 286 (blue), 133 (light green), 111 (cyan), 271 (red), 196 (yellow), 243 (dark red), 227 (dark blue).

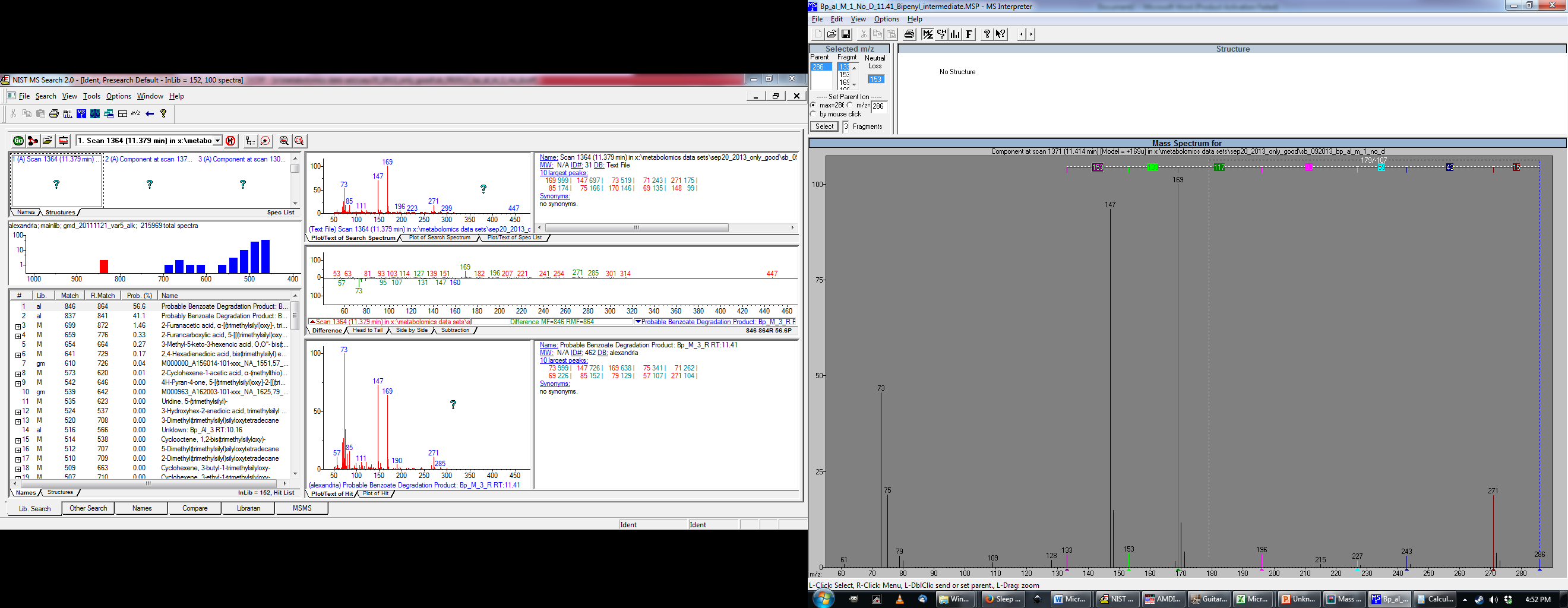


Figure 6: Mass spectrum of unknown RT 11.37.

Table 5:Functional group analysis to determine retention index of 2-hydroxymuconic semialdehyde (2TMS). Carboxyl group was interpreted as one –CO-O-.

| Group | Number of instances | Value | Sum |
| --- | --- | --- | --- |
| -CH3 | 6 | 112 | 672 |
| >Si< | 2 | -115 | -230 |
| =CH- | 3 | 102 | 306 |
| -HCO | 1 | 235 | 235 |
| =C< | 1 | 67 | 67 |
| -CO-O- | 1 | 266 | 266 |
| -O- | 1 | 75 | 75 |
| Total | x | x | 1391 |

Table 6:Functional group analysis to determine retention index of 2-hydroxymuconic semialdehyde (2TMS). Carboxyl group was interpreted as one >CO group and one –O– group.

| Group | Number of instances | Value | Sum |
| --- | --- | --- | --- |
| -CH3 | 6 | 112 | 672 |
| >Si< | 2 | -115 | -230 |
| =CH- | 3 | 102 | 306 |
| -HCO | 1 | 235 | 235 |
| =C< | 1 | 67 | 67 |
| >CO | 1 | 235 | 235 |
| -O- | 2 | 75 | 150 |
| Total | x | x | 1435 |

2-hydroxymuconic semialdehyde was drawn and derivitized to 2-hydroxymuconic semialdehyde (2TMS) (Figure 4) and fragmentation predicted (Table 4). Deconvoluted, calibrated GC spectra were searched for peaks that had all 8 predicted fragementation ions above >100. Unknown RT:11.37 was identified as containing all predicted fragments, though the adjacent, smaller peak at 11.41 also contained these ions (Figures 5 and 6). The Kovát’s retention index (Stein et al., 2007) was predicted for 2-hydroxymuconic semialdehyde (2TMS) using two possible interpretations of the functional groups present (Tables 5 and 6). The mean of these two estimates was RI:1413. This is similar to the retention index of Unknown RT:11.37 of 1557, differing by 144 units. While this is not as good a match as was observed with 2-phosphoglycolic acid, it is within range of variation of the difference between predicted and actual retention indices of compounds in the NIST library. As the mass fragmentation patterns of unknown RT:11.37 and 11.41 were very similar both were treated as possible analytes of 2-hydroxymuconic semialdehyde. Still the similarity between the predicted mass spectra of 2-hydroxymuconic semialdehyde (2TMS) and unknown RT11.37 and the predicted and actual retention index led us to conclude that unknown RT:11.37 is 2-hydroxymuconic semialdehyde (2TMS).
